# Augmentation of Transcriptomic Data for Improved Classification of Patients with Respiratory Diseases of Viral Origin

**DOI:** 10.3390/ijms23052481

**Published:** 2022-02-24

**Authors:** Magdalena Kircher, Elisa Chludzinski, Jessica Krepel, Babak Saremi, Andreas Beineke, Klaus Jung

**Affiliations:** 1 Institute for Animal Breeding and Genetics, University of Veterinary Medicine Hannover, Buenteweg 17p, 30559 Hannover, Germany; magdalena.kircher@tiho-hannover.de (M.K.); jessica.krepel@tiho-hannover.de (J.K.); babak.saremi@tiho-hannover.de (B.S.); 2 Department of Pathology, University of Veterinary Medicine Hannover, Buenteweg 17, 30559 Hannover, Germany; elisa.chludzinski@tiho-hannover.de (E.C.); andreas.beineke@tiho-hannover.de (A.B.)

**Keywords:** data augmentation, deep learning, generative adversarial networks, transcriptomic data, high-dimensional data, viral acute respiratory illness, SARS-CoV-2

## Abstract

To better understand the molecular basis of respiratory diseases of viral origin, high-throughput gene-expression data are frequently taken by means of DNA microarray or RNA-seq technology. Such data can also be useful to classify infected individuals by molecular signatures in the form of machine-learning models with genes as predictor variables. Early diagnosis of patients by molecular signatures could also contribute to better treatments. An approach that has rarely been considered for machine-learning models in the context of transcriptomics is data augmentation. For other data types it has been shown that augmentation can improve classification accuracy and prevent overfitting. Here, we compare three strategies for data augmentation of DNA microarray and RNA-seq data from two selected studies on respiratory diseases of viral origin. The first study involves samples of patients with either viral or bacterial origin of the respiratory disease, the second study involves patients with either SARS-CoV-2 or another respiratory virus as disease origin. Specifically, we reanalyze these public datasets to study whether patient classification by transcriptomic signatures can be improved when adding artificial data for training of the machine-learning models. Our comparison reveals that augmentation of transcriptomic data can improve the classification accuracy and that fewer genes are necessary as explanatory variables in the final models. We also report genes from our signatures that overlap with signatures presented in the original publications of our example data. Due to strict selection criteria, the molecular role of these genes in the context of respiratory infectious diseases is underlined.

## 1. Introduction

Viral diseases of the respiratory system are frequently studied on the molecular level of the transcriptome [1,2,3]. This can help to identify genes that are involved in the immune response or pathogenesis [4] but also to use these genes as predictors in machine-learning (ML) models to classify patients based on molecular data. Finally, ML models can help to optimize treatment strategies for patients. Facilitated by the technological progress of DNA microarrays and high-throughput RNA-sequencing (RNA-seq), molecular signatures in high-dimensional gene-expression data have been studied for over 20 years, employing classical ML techniques such as support vector machines (SVM) [5], linear discriminant analysis (LDA) [6], random forest (RF) [7], artificial neural networks (ANN) [8], or more specialized methods [9]. The term ‘molecular signature’ usually refers to a set of genes that constitute the predictor variables in an ML model. It has been shown that molecular signatures from ML models can provide ‘valuable insights into cell biology and mechanisms of human disease’ [10]. Recently, di Julio et al. [11] also studied ’transfer transcriptomic signatures’ which could have predictive value for infectious diseases across species, based on the fact that transcriptomic changes are among the early responses to infection. Such transfer signatures could therefore also be valuable to better understand the molecular processes across species. A prior step before fitting an ML model with transcriptomic data is to select a set of differentially expressed genes between two study groups (e.g., infected versus uninfected)—typically by means of statistical testing—before investigating this ‘molecular signature’ for its diagnostic or predictive power in ML models. Although differential expression analysis is usually performed on a single dataset, diagnostic or predictive signatures in an ML model are usually evaluated in independent datasets or at least by means of cross-validation. One can therefore argue that the high performance of a molecular signature in an ML model underlines the role of these genes in the molecular processes of the studied disease. Molecular processes involved in viral respiratory diseases include virus–host cell interactions, signaling pathways of the innate and adaptive immune response, as well as viral evasion strategies and tissue-repair mechanisms, which are orchestrated by the expression of specific genes. A better understanding of these pathways and the role of the respective genes is essential for the discovery of new therapeutic targets or potent prevention strategies [12,13,14].

In this work, we reanalyze two transcriptomic datasets from study contexts of respiratory, viral-caused diseases to demonstrate how data augmentation can help to improve the performance of ANNs based on high-dimensional gene-expression data. Gene-expression data for the two selected studies are freely available from the gene-expression omnibus (GEO) archive [15]. One of these two studies involves gene-expression data taken from human blood samples by means of DNA microarrays. All patients suffered from an acute respiratory illness (ARI), but with either viral or bacterial origin [16]. In the second study, gene expression was taken by means of RNA-seq and involved human nasopharyngeal swab samples from a diagnosed SARS-CoV-2 group and another group of patients with respiratory symptoms caused by different viruses such as influenza A and B, adenovirus (B, E), parainfluenza 1–4, respiratory syncytial virus A and B, human metapneumovirus, human rhinovirus, coronavirus (229E, OC43, NL63, HKU1), coxsackie/echo virus and bocavirus [8]. The authors of the original publications of the two datasets also fitted ML models to assess the diagnostic performance of selected molecular signatures, but without a step of data augmentation. Given that ML models trained with augmented versions of the original data show higher classification accuracies than without data augmentation, there would be more evidence for the important molecular role of the predictor genes involved in these models. Furthermore, ML models based on augmented data tend to be less overfitted. When being applied to draw treatment decisions, such models would result in fewer misclassifications and therefore in an improved treatment of patients.

To obtain robust ML models, a high quantity of annotated training data is usually required [17]. However, it is often difficult and expensive to obtain enough labeled samples [18], especially in the context of transcriptomics. High-dimensional gene-expression data represent expression levels for thousands of genes in a rather small number of biological samples. Thus, the dimension *d* of the data is much larger than the sample size *n*, i.e., d>>n. Furthermore, with a small sample size, it is difficult to achieve good generalization performance [19], i.e., the training data are learned well, but the trained ML model cannot easily be transferred to new independent data. In the past ten years, as ANNs have become more frequently employed for biomedical classification problems, it was found that augmented datasets can improve the classification accuracy of the trained models [20], and can also help to reduce the risk of overfitting [21]. Data augmentation means to artificially increase the sample size by modifying instances of the original data. Here, we compare three different strategies for data augmentation of high-dimensional gene-expression data. In the past few years, many algorithms based on ANNs have been developed, including the generative adversarial network (GAN), which represents a new approach to data augmentation [22]. Recent work has shown that GANs can be used successfully, among image or text generation, to add synthetic samples to insufficient training data and thus improve classification accuracy. However, it mainly addresses the problem of class imbalance [19]. Studies on enlarging relatively small datasets for classification (such as small-sized transcriptomics data) are rare [19,23]. For fitting the ANN models on the data of the two respiratory studies, we compare two variants of GAN algorithms as well as a less complex approach of mixing observations for data augmentation.

In the next section, we detail the results of the comparison of the three data augmentation techniques in the two respiratory datasets. We also compare the sets of genes in the molecular signatures from our ANN models with the signatures from the original publications. The results section is followed by a discussion about the computational and biological findings. The datasets used and the methods for data augmentation and classification are described in the last chapter.

## 2. Results

Using the two gene-expression datasets on respiratory infections, we investigated the applicability of data augmentation methods to both continuous microarray data and discrete RNA-seq count data. More specifically, we compared three different augmentation approaches: (a) a simple approach that is based on mixing observations, hereafter referred to as the mixed weighted observations (MWO) approach, as described in Section 4.6, (b) a standard GAN as described in Section 4.7, and (c) a Wasserstein GAN with gradient penalty (WGAN-GP) as described in Section 4.8. With these methods, the training datasets were expanded in such a way that initially twice as many artificial samples were added for each class as original samples were available. Evaluating the classification performance using these augmentation methods, we wanted to answer the following questions: (i) Does data augmentation increase the classification accuracy? (ii) Comparing the aforementioned methods (MWO, GAN and WGAN-GP), which one performs best for augmenting high-dimensional expression data? (iii) Can we identify genes found in the original studies, whose molecular importance is emphasized by our approach?

### 2.1. Signature Selection and Classifier Performance without Data Augmentation

We first describe the incremental signature selection for both datasets via ML without data augmentation. By increasing the number of genes, their influence on the performance of the classifier was examined. As mentioned in the introduction ANNs were used as classification model, more specifically we fitted multilayer perceptrons (MLP) with two hidden layers with 150 and 50 neurons each and rectified linear unit (ReLU) activation. The number of input neurons corresponded to the number of genes that were used as input signature. The output layer was one single neuron with linear activation. The ANN architecture is described in more detail in the methods Section 4.4.

A 10-fold cross-validation was used to estimate the performance of each classification model, i.e., the original dataset *D* was split into K=10 subsets, and for each kϵ{1,2,…,K}, the ANN was trained on the training subset D\Dk and afterwards used to predict the labels of the test subset Dk.

**Figure 1:**
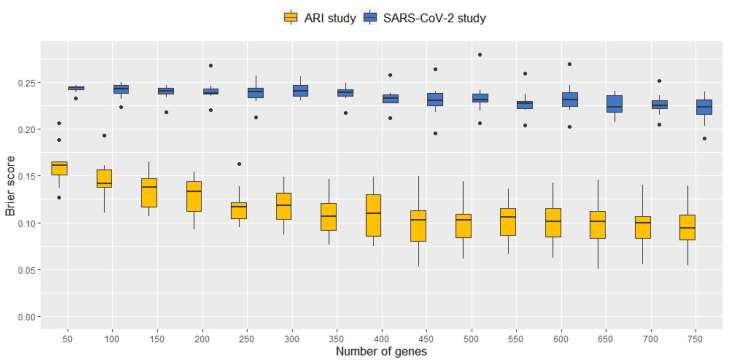
Brier scores from 10-fold cross-validation for the original microarray dataset (orange) and the original RNA-seq dataset (blue) when including an increasing number of top differentially expressed genes as input features for the ANN. The median Brier score tended to decrease when increasing the number of genes as predictors from 50 to 750.

To identify the minimum number of genes as input features required for optimal ANN classification performance, an incremental feature selection was carried out for each cross-validation training subset D\Dk. This subset was used to test the genes for differential expression and genes were ranked according to their *p*-values in ascending order, i.e., the gene with the lowest *p*-value being the first important input feature, and so on. This approach assumes that the genes with the most evidence for differential expression are also important for the discrimination of phenotypes. As a result, including an increasing number of the top ranked genes, i.e., the first 50, 100, 150, …, 750 most significant genes, as input features for training and testing the classification ANN, led to different classification performances. Figure 1 shows the distribution of the Brier score for models fit on the two respiratory datasets as obtained from the cross-validation and for different numbers of input genes. Including an increasing number of genes as input features tended to result in better performances of the ANNs, i.e., the median Brier score tended to decrease when increasing the number of top genes as input features from 50 to 750 (from 0.16 to 0.10 for the microarray data and from 0.24 to 0.22 for the RNA-seq experiment, respectively).

Performances of the ANN models for both datasets when using data augmentation are provided in the following subsections.

### 2.2. Classification Performance of Molecular Signatures Selected in the Microarray Ari Study

**Figure 2:**
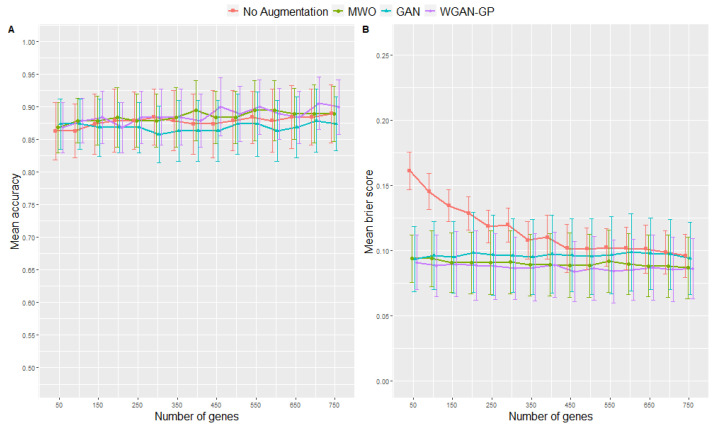
Results from 10-fold cross-validation in terms of accuracies (**A**) and Brier scores (**B**) for the microarray data when using different data augmentation methods and various numbers of top genes as input signatures. Error bars represent the 95% confidence interval of the mean.

Here, we describe the performance of ANN models trained with genes selected in the microarray study that aimed to distinguish between viral and bacterial origin of ARIs. Figure 2 shows the accuracies (left plot) and Brier scores (right plot) for the ANNs trained on different augmented datasets compared to ANNs trained without data augmentation. Numbers are also provided in Table 1 and Table 2 as well as in the Supplementary Materials. Again, the number of top genes selected as input signature was varied. Maximum accuracy, sensitivity, specificity and minimum Brier score are highlighted in each table, so that one can see the minimum number of top genes required for an optimized classification results. Looking at the Brier scores, the ANNs trained with augmented data performed clearly better than those trained without data augmentation, e.g., the lowest Brier score is obtained when using the WGAN-GP augmentation method and 450 genes. Regarding the accuracies, the WGAN-GP approach for augmenting the original training data showed the most accurate classification results by achieving the maximum mean accuracy of 0.91 when using 700 genes as input signature, followed by the MWO approach. Additionally, in terms of the Brier score, Figure 2 suggests that an increasing number of genes did not affect classification performance as much when the training data were augmented as it did with the non-augmented training data. Concerning the obtained accuracies, it seemed that the standard GAN was not performing better than the no-augmentation approach, whereas the Brier scores for the GAN approach were consistently lower than for classification using only the not-augmented original training data.

Since the above detailed differences between augmented and non-augmented scenarios describe tendencies, significant difference can also be detected using the duality between statistical test and confidence interval, as we have shown 95%—confidence intervals (CI) in all figures and tables. According to the duality between statistical test and CI, a significant difference can be inferred with a significance level of 5% when the 95%—CI for two scenarios do not overlap. Using this principle, one can see in the figures and tables clearly that data augmentation yields significantly better results in some of our simulated scenarios. Regarding the different influence of the number of genes being included in the model, from Figure 2 one can infer a significant difference between the Brier score when comparing the performances of the no-augmentation approach with either 50 or 150 top genes, whereas the CIs for the classifiers trained with augmented data do overlap.

As the previously described results of the 2-fold augmentation already led to promising results, we tested further augmentation factors, namely 1-fold, 3-fold and 5-fold augmentation. Regarding the WGAN-GP approach, it could be shown that the accuracy and the Brier score improved with increasing augmentation; an improvement in the Brier score could be observed for the MWO approach. In contrast, when increasing the number of GAN-generated samples for training, the trained classification network performed worse on the test data. The detailed results are part of the Supplementary Materials.

**Figure 3:**
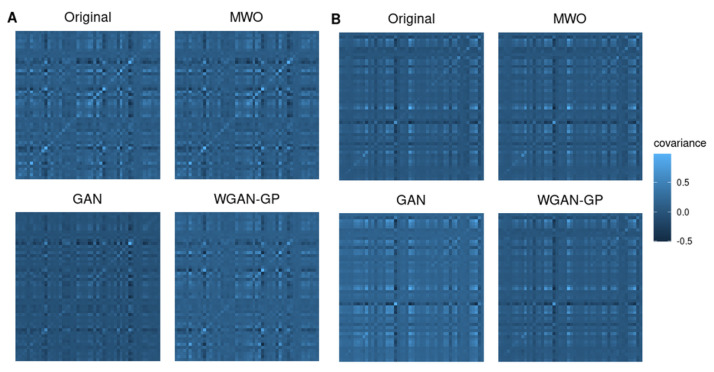
Heatmaps representing covariance matrices for selected genes from the microarray study. Covariance structures of original and artificial samples are compared for two sets of 50 randomly chosen genes. The covariance structures are best maintained when generating artificial samples with the MWO or WGAN-GP approach. The details are showed in (**A**,**B**).

Besides studying the use of data augmentation for assessing the value of a molecular signature, we were also interested how well each augmentation method maintained the correlation structure among genes. When performing data augmentation, it is important that the distribution of the artificial data closely meets that of the original data. That applies, for example, to the correlation structure between genes, which is represented, for example, by the covariance matrix of the expression matrices. Since a covariance matrix between thousands of genes cannot be estimated unbiased, we randomly selected small subsets of genes for which we studied the covariance matrices of original and artificial data. Figure 3 shows graphical representations of covariance matrices for two sets of 50 randomly selected genes from the ARI study. Comparing the covariance structures of the original data and those of the different synthetic datasets suggests that the covariance structure is maintained by each of the artificially generated samples, whereby the covariance structure seems to be most precisely maintained when generating artificial data with the MWO approach or the WGAN-GP approach. The covariance matrices of the artificial samples generated with a GAN look more different from that calculated from the original data.

**Figure 4:**
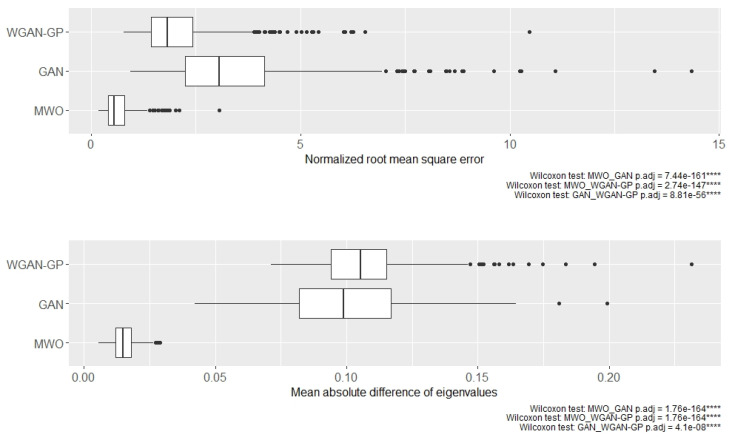
Distribution of normalized mean square errors (**top**) and mean absolute difference of eigenvalues (**bottom**) for microarray data when comparing the covariance matrices of artificial and original data. The boxplots represent data for 500 runs, in each of which 50 genes were randomly drawn and the correlation matrices were created. The symbol **** denotes *p*-value < 0.0001.

Although Figure 3 illustrates the results for two selected subsets of genes, Figure 4 shows the distribution of normalized root mean square errors and mean absolute differences for the different augmentation methods when comparing their covariance structure with the original covariance structure when 50 genes were randomly drawn, repeatedly 500 times. Comparing the three augmentation strategies, the covariance structures are best maintained when generating artificial samples with the MWO approach. Regarding the normalized root mean square error, the WGAN-GP approach shows less deviation than the GAN approach, but in terms of the mean absolute difference of eigenvalues, the WGAN-GP approach performs a bit worse than the GAN approach.

Although the training data in this example had a rather large sample size of 171, many microarray studies are much smaller. Therefore, we randomly selected a subset of 30 samples as training data, and compared again classifier performance with and without data augmentation. In this analysis, from a total of 190 samples, the 160 remaining samples were used as test samples. The whole procedure was repeated ten times to obtain confidence intervals for accuracy and the other performance measures. We compared the classification results for 2-fold and 5-fold augmentation with the classification results obtained without augmentation. Both 2-fold and 5-fold augmentation, i.e., having 30 original and 60 (150) artificial samples as training data, led to a significant improvement of the classification performance in terms of the Brier score, whereas considerable improvements of the accuracy could be observed only when including 50–200 genes into the classifier. The results for the different augmentation methods and different numbers of genes for the 2-fold and 5-fold augmentation of randomly chosen subsets are shown in the Supplementary Materials.

### 2.3. Classification Performance of Molecular Signatures Selected in the RNA-Seq SARS-CoV-2 Study

**Figure 5:**
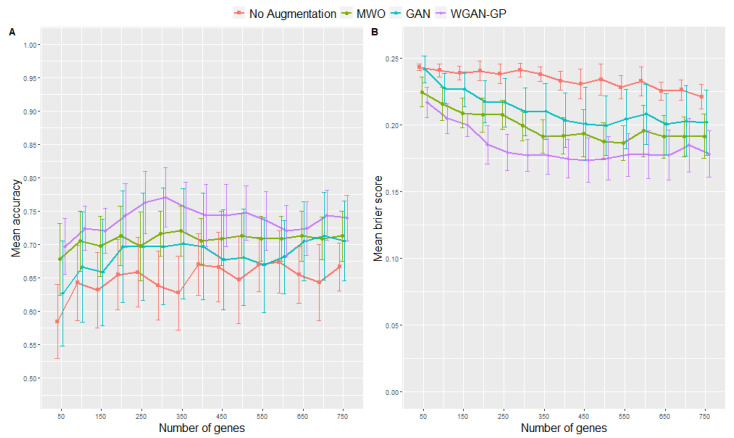
Cross validation classification accuracies (**A**) and Brier scores (**B**) for the RNA-seq experiment for different augmentation methods and various numbers of input features. Error bars represent the 95% confidence interval of the mean.

Here, we describe the performance of classification models trained with genes selected in the RNA-seq study that aimed to distinguish between SARS-CoV-2 and other viral origins of respiratory infections. Figure 5 shows the classification performances for the different augmentation strategies for the RNA-seq data. Numbers are also provided in Table 3 and Table 4 as well as in the Supplementary Materials. The accuracies (left plot) and Brier scores (right plot) for the different data augmentation methods were compared to classification without augmentation and for various numbers of genes being included as input features. Again, maximum accuracy, sensitivity, specificity and minimum Brier score are highlighted in each table, so that one can see the minimum number of top genes required for optimized classification results. E.g., in the RNA-seq example, when augmenting the original sample size by a factor of 2 using the WGAN-GP approach, the maximum accuracy of 0.77 is reached with 300 genes. However, when looking at the Brier score, 400 genes would be necessary to reach the minimum of 0.17. Regarding the Brier scores, the classification with data augmentation using the MWO or WGAN-GP approach always leads to better results than without augmentation of the training data, regardless of the number of input genes. Including the accuracy values, the WGAN-GP approach for augmenting the original training data shows the most accurate classification results, followed by the MWO approach. Regarding the obtained accuracies, it seems that the standard GAN is not performing better than the no-augmentation approach, whereas the Brier scores for the GAN approach are consistently lower than for classification using only not-augmented training data.

Again, the duality-principle between statistical test and CIs can be used to show that augmentation yields a significantly better performance of a classifier than training only with original data or between a particular number of top genes included into a classifier. One can for example infer a significant difference between the Brier score in Figure 5, when comparing the performance of the WGAN approach with either 50 or 150 of the top genes. In particular, there is no overlap between CIs of the Brier scores for classifiers trained with augmented and non-augmented data for most numbers of top genes.

As for the microarray study, also 1-fold, 3-fold and 5-fold augmentation scenarios were tested to investigate the quality of the augmented samples. With further augmentation, the results improved further for the MWO and the WGAN approach, whereas the results for the ANNs trained with GAN augmented training data were not consistently better and showed an increasing variety in the classification of the test data. Detailed results can be found in the Supplementary Materials.

**Figure 6:**
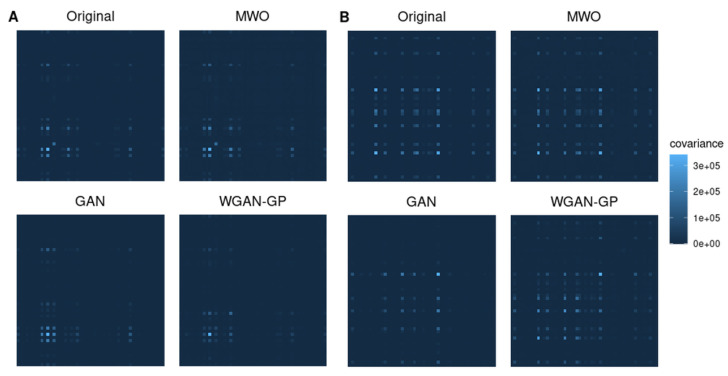
Heatmaps illustrating covariance matrices for the RNA-seq data study. Comparison of covariance matrices of original and artificial samples for two sets of 50 randomly chosen genes. The details are showed in (**A**,**B**).

Figure 6 shows again heatmaps illustrating the covariance matrices of original and artificially generated data for two sets of 50 randomly selected genes from the SARS-CoV-2 study. Comparing the covariance structures of the original data and the different synthetic data suggests that the covariance structure is mostly maintained when generating artificial data with the MWO approach. The heatmaps for the covariance matrices of the data generated with the WGAN-GP approach implies that the covariance structure is partially preserved. With the GAN approach, the covariance structure is obviously hardly preserved.

**Figure 7:**
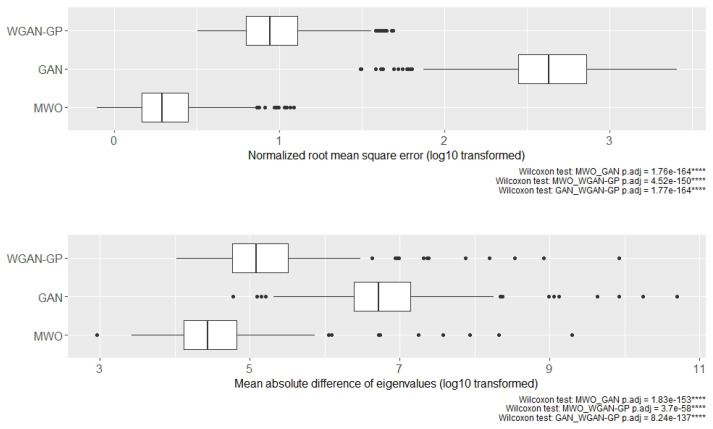
Boxplots of log-transformed normalized mean square errors (**top**) and log-transformed mean absolute differences of eigenvalues (**bottom**) for the RNA-seq experiment when comparing the covariance matrices of artificial and original data. The boxplots contain results for 500 runs, in each of which 50 genes were drawn at random and the correlation matrices were created. The symbol **** denotes *p*-value < 0.0001.

Figure 7 shows the distribution of log-transformed normalized root mean square errors and log-transformed mean absolute differences for the different augmentation methods when comparing their covariance structure with the original covariance structure when 50 genes were randomly drawn, repeatedly 500 times. In this comparison of the three augmentation strategies, the distance between the covariances of the original data and those of the artificial data generated with the MWO approach is the smallest. The corresponding deviations between the original and WGAN-GP-generated samples, on the other hand, are somewhat larger and the distance to the artificial samples that were generated with a standard GAN is the greatest.

We also studied in this RNA-seq example whether data augmentation would also be helpful with a smaller set of training data. From the 258 samples, we selected randomly a subset of 30 samples as training data, and compared again classifier performance with and without data augmentation. In this analysis, all remaining 228 samples were taken as test data. The whole procedure was repeated ten times to obtain confidence intervals for accuracy and the other performance measures. To augment the training data, we considered 2-fold augmentation and 5-fold augmentation of the small training subset. The results of the analysis support the previously shown results that augmentation improves classification performance, since here too the accuracy increased, and the Brier score decreased with increasing augmentation; for the WGAN-GP and the MWO approach more than for the GAN approach. Detailed results are part of the Supplementary Materials.

### 2.4. Molecular Signatures of Microarray and RNA-Seq Study, and Overlap with Originally Published Signatures

To select genes as molecular signatures from the microarray and RNA-seq studies, we counted in how many folds of the cross-validation a gene was selected. In each fold, the selection criterion for a gene was that the FDR-adjusted *p*-value was <0.05 and the absolute log fold change was >2. Using the number of cross-validation folds is a sharper criterion than statistical testing on the whole dataset, because multiple reduced datasets (i.e., with reduced power) are used and genes are validated in the left-out dataset.

In the microarray ARI study, 44 genes were selected in each of the 10 cross-validation runs. In the RNA-seq SARS-CoV-2 study, 88 genes were selected, accordingly. Of these 88 genes, 41 coded for small nuclear RNAs.

We compared the selected genes with those presented as signatures in the original publications of the two datasets ([8,16]). It must be said that the different ML models and different subsamples and groupings of the data were used in these publications. Hence, classifiers and selected signatures are not directly comparable. However, genes that are jointly selected in different models have more evidence of being involved in the molecular processes of the studied respiratory diseases. For the microarray study, the overlap of our signatures with the signatures presented in the originally published results was rather small, i.e., the overlap were three genes: LY6E, OASL, IFIT1. For the RNA-seq study, the overlap were 21 genes: ACAN, BATF3, BHLHA15, CACNA1F, CCL2, CCL8, CMKLR1, CST8, CXCL11, FCN1, GUK1, IFNL1, IRG1, OPRL1, OR2W5, SERPINB12, SLAMF7, TFEC, TNIP3, TRPV5, ZNF81.

Most of these genes encode for molecules that are potentially involved in the pathogenesis of viral respiratory disease, such as antiviral and interferon-associated proteins of the innate immune response (LY6E, OASL, IFIT1, IRG1, CMKLR1, FCN1), transcription factors regulating cell differentiation (BATF3), chemokines (CCL2, CCL8, CXCL11), cytokines (IFNL1), molecules of membrane receptor-ligand systems (OPRL1, OR2W5), and molecules involved in calcium pathways (BHLHA15, CACNA1F) [24,25,26,27,28,29].

Besides looking at individual genes, we also performed gene ontology (GO) enrichment analysis [30], again based on selected genes from the 10 cross-validation runs. GO terms related to biological processes (BP), molecular functions (MF) and cellular components (CC) were studied separately. Among the significant GO terms in the microarray example, only a few were related to infection or immune response, e.g., antigen processing (GO:0002483, GO:0019885). In contrast, the significant GO terms in the RNA-seq example included those related to immune response (GO:0006955), T-cell differentiation (GO:0002292), and virus or immune receptor activity (GO:0001618, GO:0140375). The detailed results of GO enrichment analysis are provided as Supplementary Tables. Further significant GO terms, which are related to infection are listed in the following:

- Microarray example, biological processes: antigen processing and presentation of endogenous peptide antigen (GO:0002483); antigen processing and presentation of endogenous peptide antigen via MHC class I (GO:0019885); antigen processing and presentation (GO:0019882); antigen processing and presentation of peptide antigen via MHC class I (GO:0002474); regulation of dendritic cell differentiation (GO:2001198); dendritic cell differentiation (GO:0097028); macrophage cytokine production (GO:0010934); antigen processing and presentation of endogenous antigen (GO:0019883); phagocytosis, engulfment (GO:0006911); antigen processing and presentation of peptide antigen (GO:0048002)
- Microarray example, cellular component: phagocytic vesicle (GO:0045335)
- Microarray example, molecular function: MHC protein binding (GO:0042287); MHC class I protein binding (GO:0042288); MHC protein complex binding (GO:0023023)
- RNA-seq example, biological processes: macrophage activation (GO:0042116); T-cell differentiation involved in immune response (GO:0002292); leukocyte activation involved in immune response (GO:0002366); regulation of macrophage activation (GO:0043030); CD4-positive, alpha-beta T-cell differentiation involved in immune response (GO:0002294); cell activation involved in immune response (GO:0002263); alpha-beta T-cell activation involved in immune response (GO:0002287); alpha-beta T-cell differentiation involved in immune response (GO:0002293); regulation of interleukin-1 production (GO:0032652); regulation of interleukin-1 beta production (GO:0032651); myeloid leukocyte activation (GO:0002274); regulation of cytokine production
  (GO:0001817); regulation of interleukin-17 production (GO:0032660); immune response (GO:0006955); T-cell activation involved in immune response (GO:0002286); response to molecule of bacterial origin (GO:0002237); cytokine production (GO:0001816); response to lipopolysaccharide (GO:0032496); cellular response to oxidative stress (GO:0034599); NIK/NF-kappaB signaling (GO:0038061); regulation of microglial cell activation (GO:1903978); T-helper cell differentiation (GO:0042093); microglial cell activation (GO:0001774); leukocyte activation involved in inflammatory response (GO:0002269); interleukin-1 production (GO:0032612); reactive oxygen species metabolic process (GO:0072593); leukocyte activation (GO:0045321); interleukin-1 beta production (GO:0032611); plasma membrane bounded cell projection assembly (GO:0120031); positive regulation of cytokine production (GO:0001819); interleukin-17 production (GO:0032620); mitochondrial translation (GO:0032543); cytokine-mediated signaling pathway (GO:0019221); CD4-positive, alpha-beta T-cell differentiation (GO:0043367); lymphocyte activation involved in immune response (GO:0002285); cell projection assembly (GO:0030031)
- RNA-seq example, molecular function: antigen binding (GO:0003823); virus receptor activity (GO:0001618); immune receptor activity (GO:0140375); cytokine receptor binding (GO:0005126); double-stranded RNA binding (GO:0003725); cytokine receptor activity (GO:0004896); chemokine binding (GO:0019956)

## 3. Discussion

Early diagnosis of patients with respiratory diseases caused by viral infections is important to provide a good treatment. Since changes in the transcriptome are among the early responses upon infection, classifiers based on high-throughput gene-expression data would provide a reasonable means for diagnosis. Early diagnosis is important to allow rapid therapeutical intervention, preventing progression of tissue damage and respiratory distress, resulting in a better prognosis. Additionally, early diagnosis is essential for the reduction of viral shedding and the initiation of early disease control strategies in the environment of affected individuals, which limits further virus spread.

Several studies have been performed to study the differentiability of patients with respiratory diseases of viral origin, usually using standard ML models fitted to transcriptomics data. We selected two of such studies to demonstrate that ANNs in combination with a step of data augmentation, can provide improved classification accuracy. Although data augmentation has a long tradition in classifying samples based on other data types (e.g., images), it has rarely been considered for transcriptomic data. We selected two different kinds of transcriptomic data, i.e., one study based on DNA microarray data and one based on RNA-seq data. Although high-dimensional gene-expression data are distributed on a continuous scale as fluorescence values from DNA microarrays [31], discrete count data are produced when using RNA-sequencing (RNA-seq) instead [32]. In summary, our comparative analyses yielded that data augmentation can indeed improve the classification performance. The best performance was achieved by an advanced generative adversarial network (WGAN-GP approach) which is, however, computationally time-consuming. The second-best method was to simply mix individual observations (MWO approach) which is computationally much less demanding.

We mainly focused on accuracy and Brier score when presenting the results. A common metric for evaluating medical diagnoses is the F1-score, which is the harmonic mean of sensitivity (recall) and positive predictive value (precision) [33]. Looking at the F1-scores supported the previous results as it behaved similar to the findings for the accuracies. i.e., for the microarray dataset no significant improvement could be observed when using all the training data for training the classifier whereas when using only a smaller subset for training at least for the first 200 most differentially expressed genes as input signature the classifiers trained with augmented data outperformed that without augmentation. For the RNA-seq data, further augmentation, i.e., increase of the augmentation factor, improved the classification both in the case of using all training data for training and the case where only a smaller subset of the data was used for training. F1-scores are also added to the Supplementary Tables and Figures.

Furthermore, our simulations have shown that the amount of augmentation has an impact on the classifier performance. In principle, the larger the amount of augmentation, the larger was the observed performance. In some cases, already with a 1-fold augmentation, a significant improvement was observed. However, a significant improvement might not necessarily mean a clinical relevant improvement. With a 5-fold augmentation in the RNA-seq example, one could obtain a significant improvement of the accuracy by 20%. This would mean a clearly larger number of correct classifications, and would be important if such a classification had an impact on therapy decisions. Therefore, it is not reasonable here to make a general statement regarding the minimum required amount of augmentation for classifiers with high-dimensional gene-expression data. Instead, we recommend checking the performance individually when other scientist use augmentation for such data.

In this manuscript, we have evaluated the use of data augmentation in the context of ANNs. Moreover, we did some additional evaluations of transcriptomic data augmentation with other classifier methods, specifically SVMs, LDA and RF, but could not see the same amount of improvement. Regarding LDA, it might be that additional observations generated by the studied approaches do not much change the estimates for group means and covariance matrices. For SVMs, additional observations generated by the studied approaches may not change much the shape of the separating hyperplane. It may be that other augmentation methods could work with SVMs, LDA and RF. Therefore, more research will be necessary to understand how augmentation of transcriptomics data can be performed in combination with different classifier models.

Independent from the question, whether augmented data can improve the classifier performance, we have compared covariance matrices of randomly selected gene sets to see, whether artificial data would also be useful for computer simulations, where researchers want to have data that reflects the correlation between genes. We found that the difference between the covariance matrix of original and artificial data was smallest for the MWO approach but larger for the GAN and WGAN-GP approaches. Although we observed these significant differences, the general shape of the covariance structure seems to be maintained as can be seen in Figure 3 and Figure 6. A reason for the observed differences with the GAN and WGAN approaches may be that with these approaches random data vectors are generated as new observations without taking the correlation structure between genes into account. For the MWO, the covariance structure is changed through the different weights with which the original columns in the gene-expression matrix are mixed. In summary, the artificial data may be useful for simulation purposes since the general shape of the covariance structure remains. Furthermore, if the covariance structure of artificial and original data was the same, the artificial data might have no added value for the classifier training. It should be added that the differences for the RNA-seq data appear much larger than the differences observed for the microarray data. This is due to the different scale of both data types.

Our classification results are not comparable with that of the ML models presented in the original publications of the two example datasets. First, we did use different groupings of samples, and second, we used other classifier models. In general, it is difficult to reimplement exactly ML models from other publications. However, our basic goal was to show that data augmentation in combination with ANNs can improve classification performance in contrast to ANNs fitted on the original data only, and that is what we have demonstrated here.

Knowledge about changes in the transcriptome during a disease can significantly contribute to a better understanding of the molecular processes of the pathogenesis and therapy response. Thus, it can be helpful to know which genes are significantly up- or down-regulated, and which GO terms are involved. We found that some of the significantly enriched GO terms have been mentioned in the context of other infectious diseases. In the microarray example, we selected the biological process GO:0002483 which was also found enriched in a study on juvenile dermatomyositis [34], which is supposed to be caused by viruses and can involve inflammation of the lung. The cellular component of phagocytic vesicles (GO:0045335) was also mentioned in the context of SARS-CoV-2 infection and pulmonary fibrosis [35]. Additionally, the enriched molecular function GO:0042287, responsible for MHC protein binding was found in a recent SARS-CoV-2 study [36], based on an enrichment analysis in 76 selected neighbor proteins of the SARS-CoV-2 spike protein. In the EBI QuickGO database the term GO:0042287 is further described as ‘Binding to a major histocompatibility complex molecule; a set of molecules displayed on cell surfaces that are responsible for lymphocyte recognition and antigen presentation’. In the RNA-seq example, the selected biological process ‘macrophage activation’ (GO:0042116) was also mentioned in the context of Asthma [37]. Furthermore, the molecular function ‘antigen binding’ (GO:00003823) was found to be enriched in a study on early-stage lung carcinoma [38].

Typically, differentially expressed genes are detected by statistical tests to judge significance and by the log fold change to quantify the strength of the effect. We have argued in the introduction that there is more evidence for the molecular importance of a set of differentially expressed genes, if these genes show additionally a high performance in a ML model. To estimate this performance more robust, we used the concept of data augmentation. In the two datasets, we have shown that the selected signatures have also a higher diagnostic power when using data augmentation for fitting the ANN models. Following our above argumentation, we think that there is good evidence regarding the molecular role of the selected set of differentially expressed genes and GO terms.

## 4. Materials and Methods

**Figure 8:**
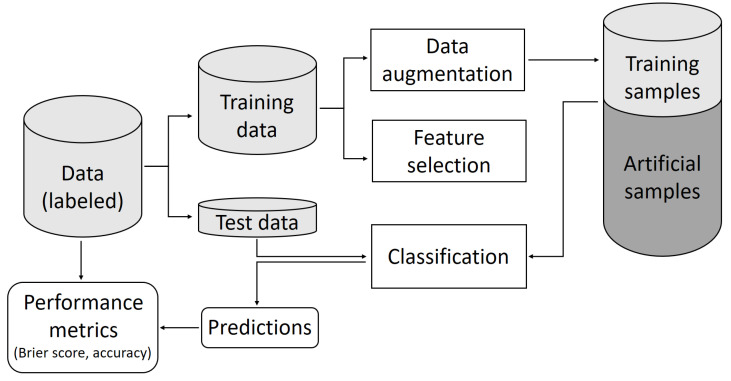
Generic pipeline: Starting at the top left the whole dataset is split into a training subset and a test subset. The training subset is then augmented using various augmentation methods. Following a feature selection, the augmented dataset with a reduced number of genes is used to train a classification network which itself is used to classify the so-far unseen test data.

We developed a standardized pipeline with varying data augmentation methods followed by training a classifier using an increasing number of preselected features and testing the classifier for unseen test data. The pipeline was performed for two real world datasets to assess the applicability of those augmentation methods and their influence on the robustness of a classification model. For the latter, we chose an artificial neural network with two hidden layers. The number of input features was successively increased, with the genes being sorted based on how strongly they are differentially expressed, i.e., incremental feature selection [39]. Two main approaches for data augmentation were taken into account: Generative adversarial networks and a less complex method as a benchmark. The number of synthetic samples generated with the different augmentation methods was two times the number of samples in each class, i.e., the class proportions have been retained. The generic pipeline is shown in Figure 8.

### 4.1. Example 1: Microarray Data from Infection Study

Data were taken from the GEO archive (https://www.ncbi.nlm.gov/geo, accessed on 15 April 2020) under the accession number GSE63990, containing microarray data of patients with respiratory infection. In the original study [16], RNA from 317 subjects with clear clinical phenotypes, namely bacterial ARI, viral ARI, non-infectious illness and healthy controls was analyzed. Total RNA was extracted from peripheral blood. The authors developed a classifier to diagnose bacterial ARI and viral ARI based on gene-expression data, finding that using patients with non-infectious illness as the control group is more appropriate than healthy individuals to identify gene-expression classifiers. For our study, we only took sequence data from individuals with viral ARI and bacterial ARI, i.e., 190 samples. No preprocessing was conducted, because the available data were already normalized based on robust multi-array average (RMA) [40] and log2 transformed.

### 4.2. Example 2: RNA-Seq Data from SARS-CoV-2 Study

Samples were taken from GEO archive (https://www.ncbi.nlm.gov/geo, accessed on 23 February 2021) under the accession number GSE163151 and contained RNA-sequencing data from nasopharyngeal swabs of humans with either SARS-CoV-2 infection, other viral ARI or non-viral ARI. Originally, the data also included whole blood samples and were used to investigate the differential host responses to SARS-CoV-2 infection [8]. For our study, we only took count data from nasopharyngeal (NP) swabs of patients with either SARS-CoV-2 infection or viral ARI, i.e., 258 samples. We conducted pre-filtering of the count data keeping only genes where at least ten samples showed at least ten counts. The filtered count data were normalized using the R package DESeq2’ [41].

### 4.3. *K*-Folds Cross-Validation

*K*-folds cross-validation was used to evaluate the different augmentation methods. *K*-folds cross-validation is a common approach for estimating the accuracy of a classifier, and for model selection [42]. Therefore, the data *D* is randomly split into *K* non-overlapping subsets (folds) $D_{1}$, $D_{2}$, …, DK of approximately equal size. The algorithm is therefore trained and validated *K* times: for each kϵ{1,2,…,K}, it is trained on D\Dk and validated on Dk [43]. In our study, we split the respective training data into *K* = 10 subsets preserving the percentage of samples per class (i.e., stratified *K*-folds cross-validation [42]) by a single split of the data before starting the training. Beginning with the first fold as test set, feature selection, data augmentation and training of the classifier is conducted for the respective training subset. The trained network is then used to predict the labels (outcome) of the so-far unseen test data. Performance metrics including accuracy and Brier score are calculated for each fold and stored for later comparisons. Although the accuracy is the proportion of correctly classified samples of all samples, the Brier score measures the mean squared difference between the probability of belonging to class 1 and the actual outcome of the sample (0 or 1):

Let $y_{i}\;\epsilon \;\left\{0,1\right\}$ be the outcome of the event for sample i. Transforming the output of the classification ANN as described in Section 4.4 by means of the logistic sigmoid function, i.e., $S\left(x_{i}\right)=\frac{e^{x_{i}}}{e^{x_{i}}+1}$ with $x_{i}$ being the output value of the classification ANN for sample i, the latter is mapped to a value between zero and one. This can be interpreted as the estimated probability that sample *i* has the outcome 1, denoted by $\hat{p}\left(y_{i}=1\right)$. The predicted outcome is assigned as follows:

(1) $$\hat{y_{i}}=\left\{\begin{array}{cc} 0, & \mathrm{if}\;\hat{p}\left(y_{i}=1\right)\leq 0.5\\ 1, & \mathrm{if}\;\hat{p}\left(y_{i}=1\right)>0.5 \end{array}\right.$$

The proportion of correctly classified samples, i.e., $\hat{y_{i}}\;==\;y_{i}$, of all samples, is defined as the accuracy.

In contrast, the Brier score measures the mean squared difference between the probability of belonging to class 1 and the actual outcome of the sample [44]:

(2) $$Brier\;score=\frac{1}{N}\sum_{i=1}^{N}{\left(y_{i}-\hat{p}\left(y_{i}=1\right)\right)}^{2}$$

Thus, the Brier score provides a more detailed measure for the performance of a classifier than the accuracy.

### 4.4. Classification with Artificial Neural Networks

For classification, a deep neural network with two hidden layers with 150 and 50 neurons each was used. The number of input neurons corresponded to the number of input features (genes). In the hidden layers, rectified linear unit activation (ReLU) function was used to transform the input values, i.e., the output of the respective previous layer. ReLU function is a common type of non-linear activation used in hidden layers that maps negative values to zero and for positive values the function returns the input value [45]. The output layer was one single neuron with linear activation. As the criterion for backward propagation, binary crossentropy with logits loss, which is a combination of a sigmoid layer and binary cross entropy loss in one single class, was used. Stochastic gradient descent algorithm was used to optimize the height of the weights, i.e., to minimize the classification loss. Dropout regularization, i.e., random selection of neurons that are set to zero, was used to reduce overfitting [46]. The neural network was built using the open-source program library PyTorch ([47]) and the PyTorch research framework PyTorch Lightning [48].

### 4.5. Incremental Feature Selection

The number of input features that were passed to the classification ANN was successively increased, with those features being sorted accordingly to their importance, i.e., the most differentially expressed genes. Therefore, a feature selection was carried out for each cross-validation fold and accordingly for each training subset, and the classification network was trained and tested with different numbers of those preselected genes. For both microarray data and RNA-seq data, the selection of genes followed a test for differential expression which was conducted using the R packages ‘Limma’ [49] and ‘DESeq2’ [41], respectively. *p*-values were obtained for gene ranking and the resulting lists of ranked genes were used to extract the top *n* genes.

### 4.6. Augmentation by Weighted Mixed Observations

The approach to augment data by weighted mixed observations is mainly used for image data augmentation and is described by Shorten et al. [21] as mixing images. Similarly, we generated new samples by matrix multiplication. The original data, represented by a *p* × *n* matrix, with *p* being the number of features and *n* being the number of samples is multiplied by a (*n* × 1) vector of randomly chosen small weights. Repeating this calculation $n^{*}$ times, each time with a randomly drawn weights vector and appending the vectors generated in this way, results in a $n^{*}$ × *p* matrix, with $n^{*}$ being the required number of artificial samples. *Z*-transforming that matrix, we obtain a synthetic dataset consisting of the new generated arrays that lie within the range of the original data.

### 4.7. Augmentation by Gan

A generative adversarial network (GAN) is a type of generative model based on deep learning [50] which was introduced to the machine-learning community in 2014 by Ian J. Goodfellow [22]. Unlike the supervised deep learning method, we use for classification, GAN models belong to unsupervised learning, i.e., no labels are given to train the model [51]. GANs have been widely used for image datasets and are continually developing. However, to our knowledge, only a few attempts have been made to use GANs for augmentation of gene-expression datasets [23,52,53].

A conventional GAN consists of two deep neural networks which are embedded in a competitive process. The discriminator works as a classifier and discriminates between real and fake samples. The generator generates synthetic samples from a latent vector *z* to degrade the classification performance of the discriminator, i.e., to maximize the cost of the discriminator [17]. As suggested by Goodfellow et al. [22], the discriminator is trained for *c* epochs to minimize its loss before the generator is trained for one epoch. Through this competition, the model theoretically learns a generator that creates realistic data, i.e., that samples from the complex, high-dimensional training distribution [22]. Viñas et al. [52] point to a problem with the augmentation of gene-expression data, namely the difficulty of recognizing whether the gene-expression data generated by the GAN is realistic, which is a subordinate problem in other domains such as image generation, where it can be determined by empirical investigation. Essentially, the objective of the GAN is to minimize the so-called Jensen–Shannon Divergence (JSD), which itself is a metric for the similarity between two probability distributions [22].

In our model, the GAN consisted of a generator, which in turn was a MLP with three hidden layers with 250, 500 and 1000 neurons each with ReLU activation, and a discriminator, which was also a MLP with three hidden layers with 1000, 500 and 250 neurons each with leaky ReLU activation. The generator takes a latent vector *z*, consisting of 100 randomly selected values between 0 and 1 from the normal distribution, processes it through the hidden layers and maps it onto a high-dimensional vector that describes an artificial sample. Thus, the generator has as many output neurons as the number of genes that make up an original sample, each with hyperbolic tangent (Tanh) activation. The discriminator takes such a vector as input vector, i.e., its number of input features corresponds to the number of output neurons of the generator, processes it through the hidden layers and maps it to a single output neuron with linear activation. Binary crossentropy with logits loss was used as the criterion for backward propagation for both discriminator and generator. Adam optimization algorithm [54] with a learning rate of 0.0001 was used to optimize the height of the weights of the discriminator ANN and the generator ANN. Training the GAN started with training of the discriminator network for one time, followed by training of the generator once as well. After training the GAN accordingly for 2000 epochs, a $n^{*}$ × 100 matrix, consisting of $n^{*}$ 100-dimensional noise vectors each containing values that are randomly sampled from gaussian distribution, was passed to the trained generator to generate $n^{*}$ artificial samples. The GAN was built using PyTorch [47].

### 4.8. Augmentation by Wasserstein Gan with Gradient Penalty

As one problem with GANs is their training instability [55], much of the recent work on GANs is on suggesting variants of the classical GAN architecture to stabilize the training process. One alternative is the Wasserstein GAN (WGAN) proposed by Arjovski et al. [55], which uses the Earth-Mover (Wasserstein) distance instead of Jensen–Shannon divergence (JSD) to measure the closeness of the modelled probability distribution and the real probability distribution. Earth-Mover distance has its roots in transport theory and is, in simple terms, the ‘cost of the optimal transport plan’ [55] to transform one distribution into another. Compared to JSD, the Wasserstein Distance has the advantage that it is always continuous and has an exploitable gradient almost everywhere, which makes it a more sensible cost function if the real data distribution and the parametric probability distribution do not share the support or do not overlap, i.e., when the distributions are disjoint [55,56]. However, the Wasserstein GAN which is based on the Kantorovich-Rubinstein duality of Wasserstein distance, brings an additional Lipschitz requirement, i.e., the discriminator must lie within the space of 1-Lipschitz functions [55]. In the standard WGAN [55], this constraint is implemented using weight clipping. Instead of the latter, Gulrajani et al. [57] propose the use of Wasserstein GAN with gradient penalty, i.e., constraining the gradient norm of the critic with respect to its input, which showed better performance than standard GAN or standard WGAN.

In our study, we employed a Wasserstein GAN with gradient penalty (WGAN-GP). For this we used the same architecture for the generator ANN and the discriminator ANN as with the GAN, but changed the loss function by adding a gradient penalty. For implementing the gradient penalty term, we modified code from Persson [58], who wrote a PyTorch script for a WGAN-GP based on the suggestions of Arjovski et al. [55] and Guljarani et al. [57].

## Figures and Tables

**Table 1:** Classification performance measures for microarray data (test subsets) after training with original training subset only. The table shows the 95% confidence intervals (mean ± 1.96 standard errors) for accuracy, Brier score, sensitivity and specificity, respectively. Values in bold are the best scores for the respective performance metric.

Method	Number of Top Genes	Accuracy	Brier Score	Sensitivity	Specificity
No	50	0.86 ± 0.0440	0.16 ± 0.0143	0.92 ± 0.0515	0.76 ± 0.0998
Augmentation	100	0.86 ± 0.0413	0.15 ± 0.0140	0.92 ± 0.0487	0.78 ± 0.1090
	150	0.87 ± 0.0466	0.13 ± 0.0121	0.92 ± 0.0514	0.79 ± 0.1090
	200	0.88 ± 0.0488	0.13 ± 0.0128	**0.93 ± 0.0535**	0.79 ± 0.1090
	250	0.88 ± 0.0436	0.12 ± 0.0124	0.93 ± 0.0411	0.79 ± 0.1037
	300	0.88 ± 0.0429	0.12 ± 0.0129	0.92 ± 0.0514	0.82 ± 0.0887
	350	0.88 ± 0.0463	0.11 ± 0.0144	0.92 ± 0.0515	0.81 ± 0.0939
	400	0.87 ± 0.0466	0.11 ± 0.0168	0.92 ± 0.0487	0.81 ± 0.1028
	450	0.87 ± 0.0515	0.10 ± 0.0184	0.92 ± 0.0515	0.79 ± 0.1118
	500	0.88 ± 0.0463	0.10 ± 0.0160	0.92 ± 0.0514	0.81 ± 0.0998
	550	0.88 ± 0.0401	0.10 ± 0.0145	0.92 ± 0.0487	**0.84 ± 0.0748**
	600	0.88 ± 0.0488	0.10 ± 0.0164	0.92 ± 0.0515	0.81 ± 0.0939
	650	0.88 ± 0.0481	0.10 ± 0.0184	0.92 ± 0.0515	0.82 ± 0.0919
	700	0.88 ± 0.0429	0.10 ± 0.0168	0.92 ± 0.0515	0.82 ± 0.0783
	750	**0.89 ± 0.0447**	**0.10 ± 0.0166**	0.93 ± 0.0411	0.82 ± 0.0919

**Table 2:** Classification performance measures for microarray data (test subsets) after training with augmented training data either augmented with mixed weighted observations (‘MWO’), a standard generative adversarial network (‘GAN’) or the advanced Wasserstein GAN with gradient penalty (‘WGAN-GP’) with the number of synthetic samples being two times the number of original samples in each class, respectively. The table shows the 95% confidence intervals (mean ± 1.96 standard errors) for accuracy, Brier score, sensitivity and specificity, respectively. Values in bold are the best scores for the respective performance metric.

Method	Number of Top Genes	Accuracy	Brier Score	Sensitivity	Specificity
MWO	50	0.87 ± 0.0384	0.09 ± 0.0181	0.91 ± 0.0451	0.81 ± 0.0805
	100	0.88 ± 0.0346	0.09 ± 0.0216	0.91 ± 0.0451	0.83 ± 0.0798
	150	0.88 ± 0.0378	0.09 ± 0.0228	0.92 ± 0.0487	0.82 ± 0.0783
	200	0.88 ± 0.0456	0.09 ± 0.0235	0.92 ± 0.0487	0.84 ± 0.0953
	250	0.88 ± 0.0408	0.09 ± 0.0245	0.91 ± 0.0451	0.84 ± 0.0953
	300	0.88 ± 0.0408	0.09 ± 0.0243	0.91 ± 0.0451	0.84 ± 0.0953
	350	0.88 ± 0.0456	0.09 ± 0.0236	0.92 ± 0.0487	0.84 ± 0.0953
	400	0.89 ± 0.0461	0.09 ± 0.0239	0.92 ± 0.0487	0.86 ± 0.0760
	450	0.88 ± 0.0401	0.09 ± 0.0247	0.92 ± 0.0487	0.84 ± 0.0748
	500	0.88 ± 0.0401	0.09 ± 0.0242	0.91 ± 0.0451	0.85 ± 0.0815
	550	0.89 ± 0.0461	0.09 ± 0.0240	0.92 ± 0.0487	0.86 ± 0.0760
	600	0.89 ± 0.0461	0.09 ± 0.0235	0.92 ± 0.0487	0.86 ± 0.0760
	650	0.89 ± 0.0391	0.09 ± 0.0238	0.91 ± 0.0451	0.86 ± 0.0760
	700	0.89 ± 0.0447	0.09 ± 0.0240	0.92 ± 0.0487	0.85 ± 0.0815
	750	0.89 ± 0.0420	0.09 ± 0.0237	0.91 ± 0.0451	0.86 ± 0.0760
GAN	50	0.87 ± 0.0383	0.09 ± 0.0248	0.9 ± 0.0484	0.84 ± 0.0748
	100	0.87 ± 0.0383	0.10 ± 0.0261	0.90 ± 0.0484	0.84 ± 0.0748
	150	0.87 ± 0.0442	0.09 ± 0.0275	0.90 ± 0.0484	0.82 ± 0.0919
	200	0.87 ± 0.0384	0.10 ± 0.0307	0.89 ± 0.0497	0.84 ± 0.0748
	250	0.87 ± 0.0384	0.10 ± 0.0308	0.89 ± 0.0497	0.84 ± 0.0748
	300	0.86 ± 0.0436	0.10 ± 0.0283	0.88 ± 0.0504	0.82 ± 0.0919
	350	0.86 ± 0.0466	0.10 ± 0.0286	0.88 ± 0.0504	0.84 ± 0.0889
	400	0.86 ± 0.0466	0.10 ± 0.0297	0.88 ± 0.0504	0.84 ± 0.0889
	450	0.86 ± 0.0466	0.10 ± 0.0280	0.87 ± 0.0561	0.85 ± 0.0848
	500	0.87 ± 0.0466	0.10 ± 0.0291	0.88 ± 0.0504	0.87 ± 0.0897
	550	0.87 ± 0.0491	0.10 ± 0.0298	0.89 ± 0.0497	0.85 ± 0.0945
	600	0.86 ± 0.0466	0.10 ± 0.0297	0.88 ± 0.0504	0.84 ± 0.0889
	650	0.87 ± 0.0468	0.10 ± 0.0275	0.88 ± 0.0504	0.85 ± 0.0848
	700	0.88 ± 0.0488	0.10 ± 0.0270	0.89 ± 0.0497	0.87 ± 0.0897
	750	0.87 ± 0.0413	0.09 ± 0.0277	0.88 ± 0.0560	0.86 ± 0.0635
WGAN-GP	50	0.87 ± 0.0384	0.09 ± 0.021	0.92 ± 0.0487	0.79 ± 0.0916
	100	0.88 ± 0.0346	0.09 ± 0.0235	0.91 ± 0.0451	0.83 ± 0.0798
	150	0.88 ± 0.0401	0.09 ± 0.0249	0.92 ± 0.0487	0.84 ± 0.0748
	200	0.87 ± 0.0384	0.09 ± 0.0264	0.91 ± 0.0451	0.81 ± 0.0906
	250	0.88 ± 0.0401	0.09 ± 0.0254	0.91 ± 0.0451	0.85 ± 0.0815
	300	0.88 ± 0.0429	0.09 ± 0.0240	0.92 ± 0.0487	0.84 ± 0.0953
	350	0.88 ± 0.0429	0.09 ± 0.0260	0.92 ± 0.0487	0.84 ± 0.0953
	400	0.88 ± 0.0408	0.09 ± 0.0250	0.91 ± 0.0451	0.84 ± 0.0953
	450	0.90 ± 0.0447	**0.08 ± 0.0231**	**0.93 ± 0.0535**	0.85 ± 0.0862
	500	0.89 ± 0.0420	0.09 ± 0.0245	0.92 ± 0.0487	0.85 ± 0.0815
	550	0.90 ± 0.0420	0.08 ± 0.0241	0.92 ± 0.0487	**0.88 ± 0.0688**
	600	0.89 ± 0.0391	0.09 ± 0.0235	0.92 ± 0.0487	0.85 ± 0.0862
	650	0.88 ± 0.0401	0.09 ± 0.0248	0.91 ± 0.0451	0.85 ± 0.0916
	700	**0.91 ± 0.0401**	0.09 ± 0.0247	0.92 ± 0.0514	0.88 ± 0.0618
	750	0.90 ± 0.0420	0.09 ± 0.0231	0.92 ± 0.0514	0.86 ± 0.0696

**Table 3:** Classification performance measures for RNA-seq data (test subsets) after training with original training data only. The table shows the 95% confidence intervals (mean ± 1.96 standard errors) for accuracy, Brier score, sensitivity and specificity, respectively. Values in bold are the best scores for the respective performance metric.

Method	Number of Top Genes	Accuracy	Brier Score	Sensitivity	Specificity
No	50	0.59 ±0.0557	0.24 ±0.0027	0.24 ±0.1100	**0.98 ±0.0218**
Augmentation	100	0.64 ± 0.0564	0.24 ± 0.0050	0.52 ± 0.1806	0.79 ± 0.2591
	150	0.63 ± 0.0563	0.24 ± 0.0051	0.43 ± 0.1501	0.86 ± 0.1902
	200	0.66 ± 0.0527	0.24 ± 0.0073	0.43 ± 0.0976	0.92 ± 0.0544
	250	0.66 ± 0.0524	0.24 ± 0.0074	0.54 ± 0.1211	0.79 ± 0.1842
	300	0.64 ± 0.0512	0.24 ± 0.0051	0.54 ± 0.1332	0.76 ± 0.1813
	350	0.63 ± 0.0550	0.24 ± 0.0056	0.50 ± 0.1405	0.78 ± 0.1825
	400	0.67 ± 0.0464	0.23 ± 0.0072	0.51 ± 0.0720	0.86 ± 0.049
	450	0.67 ± 0.0518	0.23 ± 0.0109	**0.57 ± 0.1160**	0.78 ± 0.1652
	500	0.65 ± 0.0657	0.23 ± 0.0117	0.46 ± 0.1185	0.86 ± 0.0845
	550	0.67 ± 0.0411	0.23 ± 0.0086	0.54 ± 0.0630	0.82 ± 0.0722
	600	**0.67 ± 0.0469**	0.23 ± 0.0110	0.54 ± 0.0663	0.82 ± 0.0514
	650	0.66 ± 0.0425	0.23 ± 0.0069	0.53 ± 0.0516	0.80 ± 0.0697
	700	0.64 ± 0.0570	0.23 ± 0.0078	0.52 ± 0.0482	0.78 ± 0.1222
	750	0.67 ± 0.0355	**0.22 ± 0.0094**	0.54 ± 0.0547	0.81 ± 0.0490

**Table 4:** Classification performance measures for RNA-seq data (test subsets) after training with augmented training data either augmented with mixed weighted observations (‘MWO’), a standard generative adversarial network (‘GAN’) or the advanced Wasserstein GAN with gradient penalty (‘WGAN-GP’) with the number of synthetic samples being two times the number of original samples in each class, respectively. The table shows the 95% confidence intervals (mean ± 1.96 standard errors) for accuracy, Brier score, sensitivity and specificity, respectively. Values in bold are the best scores for the respective performance metric.

Method	Number of Top Genes	Accuracy	Brier Score	Sensitivity	Specificity
MWO	50	0.68 ± 0.0533	0.22 ± 0.0111	0.43 ± 0.0933	**0.96 ± 0.0365**
	100	0.71 ± 0.0450	0.22 ± 0.0125	0.52 ± 0.0654	0.92 ± 0.0544
	150	0.70 ± 0.0448	0.21 ± 0.0109	0.53 ± 0.0729	0.89 ± 0.0691
	200	0.71 ± 0.0447	0.21 ± 0.0126	0.56 ± 0.0651	0.89 ± 0.0547
	250	0.70 ± 0.0511	0.21 ± 0.0111	0.56 ± 0.0595	0.86 ± 0.0772
	300	0.72 ± 0.0338	0.20 ± 0.0112	0.58 ± 0.0481	0.88 ± 0.0609
	350	0.72 ± 0.0374	0.19 ± 0.0126	0.59 ± 0.0505	0.87 ± 0.0606
	400	0.71 ± 0.0341	0.19 ± 0.0136	0.60 ± 0.0466	0.82 ± 0.0618
	450	0.71 ± 0.0405	0.19 ± 0.0175	0.62 ± 0.0480	0.81 ± 0.0732
	500	0.71 ± 0.0338	0.19 ± 0.0139	0.62 ± 0.0537	0.82 ± 0.0618
	550	0.71 ± 0.0338	0.19 ± 0.0131	0.62 ± 0.0487	0.81 ± 0.0425
	600	0.71 ± 0.0338	0.20 ± 0.0194	0.64 ± 0.0406	0.79 ± 0.0609
	650	0.71 ± 0.0373	0.19 ± 0.0160	0.63 ± 0.0475	0.81 ± 0.0547
	700	0.71 ± 0.0318	0.19 ± 0.0151	0.63 ± 0.0518	0.80 ± 0.0499
	750	0.71 ± 0.0373	0.19 ± 0.0165	0.63 ± 0.0475	0.81 ± 0.0547
GAN	50	0.63 ± 0.0792	0.24 ± 0.0102	0.76 ± 0.1749	0.48 ± 0.3068
	100	0.67 ± 0.0828	0.23 ± 0.0113	0.78 ± 0.1292	0.53 ± 0.2768
	150	0.66 ± 0.0794	0.23 ± 0.0131	0.80 ± 0.1247	0.50 ± 0.2700
	200	0.70 ± 0.0836	0.22 ± 0.0157	0.86 ± 0.0934	0.52 ± 0.2481
	250	0.70 ± 0.0805	0.22 ± 0.0183	0.87 ± 0.0776	0.50 ± 0.2435
	300	0.70 ± 0.0873	0.21 ± 0.0178	0.87 ± 0.0974	0.50 ± 0.2447
	350	0.70 ± 0.0823	0.21 ± 0.0207	0.89 ± 0.0840	0.49 ± 0.2354
	400	0.70 ± 0.0798	0.20 ± 0.0202	0.89 ± 0.0840	0.48 ± 0.2320
	450	0.68 ± 0.0747	0.20 ± 0.0273	0.89 ± 0.0760	0.43 ± 0.2294
	500	0.68 ± 0.0724	0.20 ± 0.0222	0.89 ± 0.0760	0.44 ± 0.2259
	550	0.67 ± 0.0712	0.20 ± 0.0225	0.88 ± 0.0862	0.43 ± 0.2242
	600	0.68 ± 0.0550	0.21 ± 0.0225	0.89 ± 0.0814	0.45 ± 0.1936
	650	0.71 ± 0.0591	0.20 ± 0.0228	0.89 ± 0.0760	0.49 ± 0.1909
	700	0.71 ± 0.0653	0.20 ± 0.0262	**0.91 ± 0.0628**	0.49 ± 0.2029
	750	0.71 ± 0.0597	0.20 ± 0.0244	0.90 ± 0.0633	0.48 ± 0.1802
WGAN-GP	50	0.70 ± 0.0418	0.22 ± 0.0114	0.48 ± 0.0801	0.95 ± 0.0436
	100	0.72 ± 0.0333	0.20 ± 0.0111	0.56 ± 0.0367	0.92 ± 0.0544
	150	0.72 ± 0.0334	0.20 ± 0.0088	0.59 ± 0.0398	0.88 ± 0.0609
	200	0.74 ± 0.0474	0.18 ± 0.0143	0.65 ± 0.0580	0.85 ± 0.0722
	250	0.76 ± 0.0462	0.18 ± 0.0137	0.68 ± 0.0605	0.86 ± 0.0646
	300	**0.77 ± 0.0448**	0.18 ± 0.0120	0.70 ± 0.0572	0.86 ± 0.0691
	350	0.76 ± 0.0379	0.18 ± 0.0143	0.70 ± 0.0533	0.83 ± 0.0568
	400	0.74 ± 0.0469	0.17 ± 0.0146	0.70 ± 0.0644	0.80 ± 0.0606
	450	0.74 ± 0.0469	**0.17 ± 0.0165**	0.71 ± 0.0584	0.78 ± 0.0697
	500	0.75 ± 0.0404	0.17 ± 0.0165	0.72 ± 0.0482	0.78 ± 0.0653
	550	0.74 ± 0.0440	0.18 ± 0.0160	0.72 ± 0.0500	0.75 ± 0.0689
	600	0.72 ± 0.0385	0.18 ± 0.0177	0.70 ± 0.0490	0.75 ± 0.0596
	650	0.72 ± 0.0402	0.18 ± 0.0187	0.70 ± 0.0561	0.75 ± 0.0596
	700	0.74 ± 0.0373	0.18 ± 0.0200	0.75 ± 0.0668	0.74 ± 0.0665
	750	0.74 ± 0.0344	0.18 ± 0.0173	0.74 ± 0.0472	0.74 ± 0.0618

## Data Availability

The data presented in this study are openly available in Gene-Expression Omnibus Archive (https://www.ncbi.nlm.gov/geo, accessed on 15 April 2020) under the accession numbers GSE63990 and GSE163151. No new data were created or analyzed in this study. Data sharing is not applicable to this article.

## Supplementary Materials

The following are available online at https://www.mdpi.com/article/10.3390/ijms23052481/s1.

## Abbreviations

The following abbreviations are used in this manuscript:

ANN	Artificial neural network
ARI	Acute respiratory illness
CI	Confidence interval
GAN	Generative adversarial network
GEO	Gene-expression omnibus
GO	Gene ontology
ML	Machine learning
MWO	Mixed weighted observations
ReLU	Rectified linear unit
WGAN	Wasserstein generative adversarial network
WGAN-GP	Wasserstein generative adversarial network with gradient penalty
