# Supplementary material for: Augmentation of Transcriptomic Data for Improved Classification of Patients with Respiratory Diseases of Viral Origin
Source: Int J Mol Sci. 2022 Feb 24;23(5):2481. doi: 10.3390/ijms23052481 (PMC8910329; doi:10.3390/ijms23052481)
Supplement: Supplementary file 1 [file ijms-23-02481-s001.zip › SupplementaryFigures.pptx]

## Slide 1
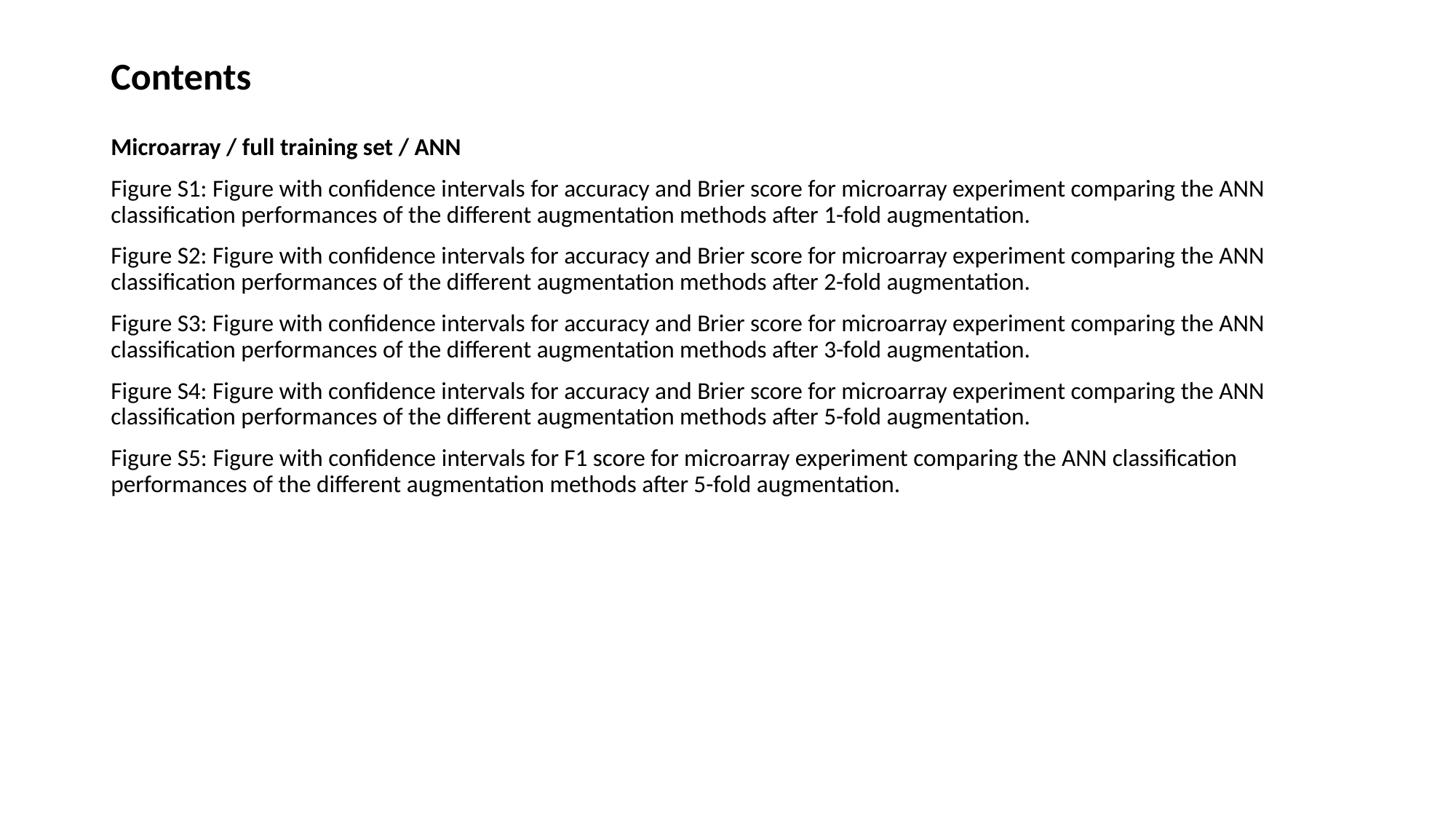

# Contents
Microarray / full training set / ANN
Figure S1: Figure with confidence intervals for accuracy and Brier score for microarray experiment comparing the ANN classification performances of the different augmentation methods after 1-fold augmentation.
Figure S2: Figure with confidence intervals for accuracy and Brier score for microarray experiment comparing the ANN classification performances of the different augmentation methods after 2-fold augmentation.
Figure S3: Figure with confidence intervals for accuracy and Brier score for microarray experiment comparing the ANN classification performances of the different augmentation methods after 3-fold augmentation.
Figure S4: Figure with confidence intervals for accuracy and Brier score for microarray experiment comparing the ANN classification performances of the different augmentation methods after 5-fold augmentation.
Figure S5: Figure with confidence intervals for F1 score for microarray experiment comparing the ANN classification performances of the different augmentation methods after 5-fold augmentation.

## Slide 2
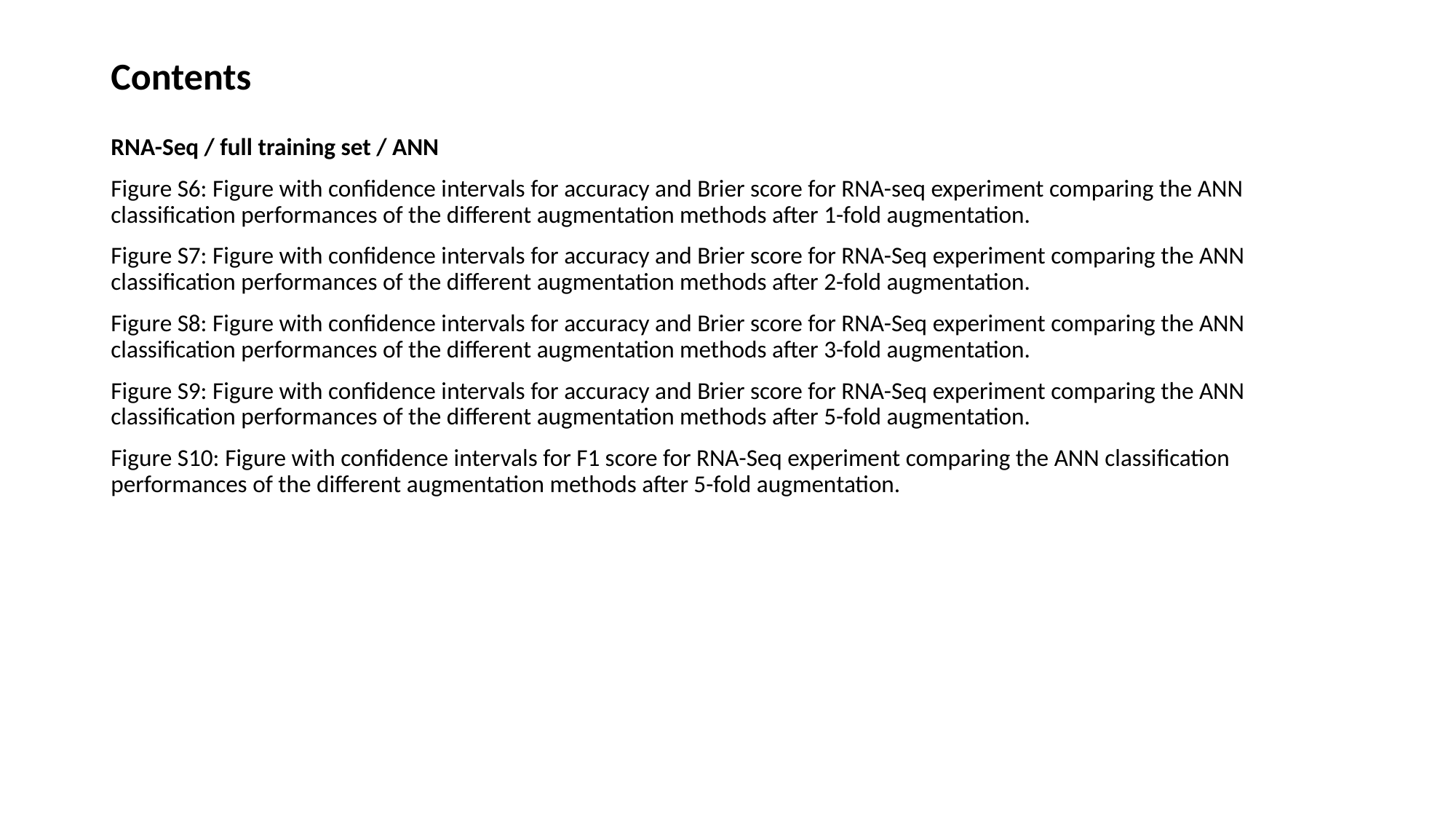

# Contents
RNA-Seq / full training set / ANN
Figure S6: Figure with confidence intervals for accuracy and Brier score for RNA-seq experiment comparing the ANN classification performances of the different augmentation methods after 1-fold augmentation.
Figure S7: Figure with confidence intervals for accuracy and Brier score for RNA-Seq experiment comparing the ANN classification performances of the different augmentation methods after 2-fold augmentation.
Figure S8: Figure with confidence intervals for accuracy and Brier score for RNA-Seq experiment comparing the ANN classification performances of the different augmentation methods after 3-fold augmentation.
Figure S9: Figure with confidence intervals for accuracy and Brier score for RNA-Seq experiment comparing the ANN classification performances of the different augmentation methods after 5-fold augmentation.
Figure S10: Figure with confidence intervals for F1 score for RNA-Seq experiment comparing the ANN classification performances of the different augmentation methods after 5-fold augmentation.

## Slide 3
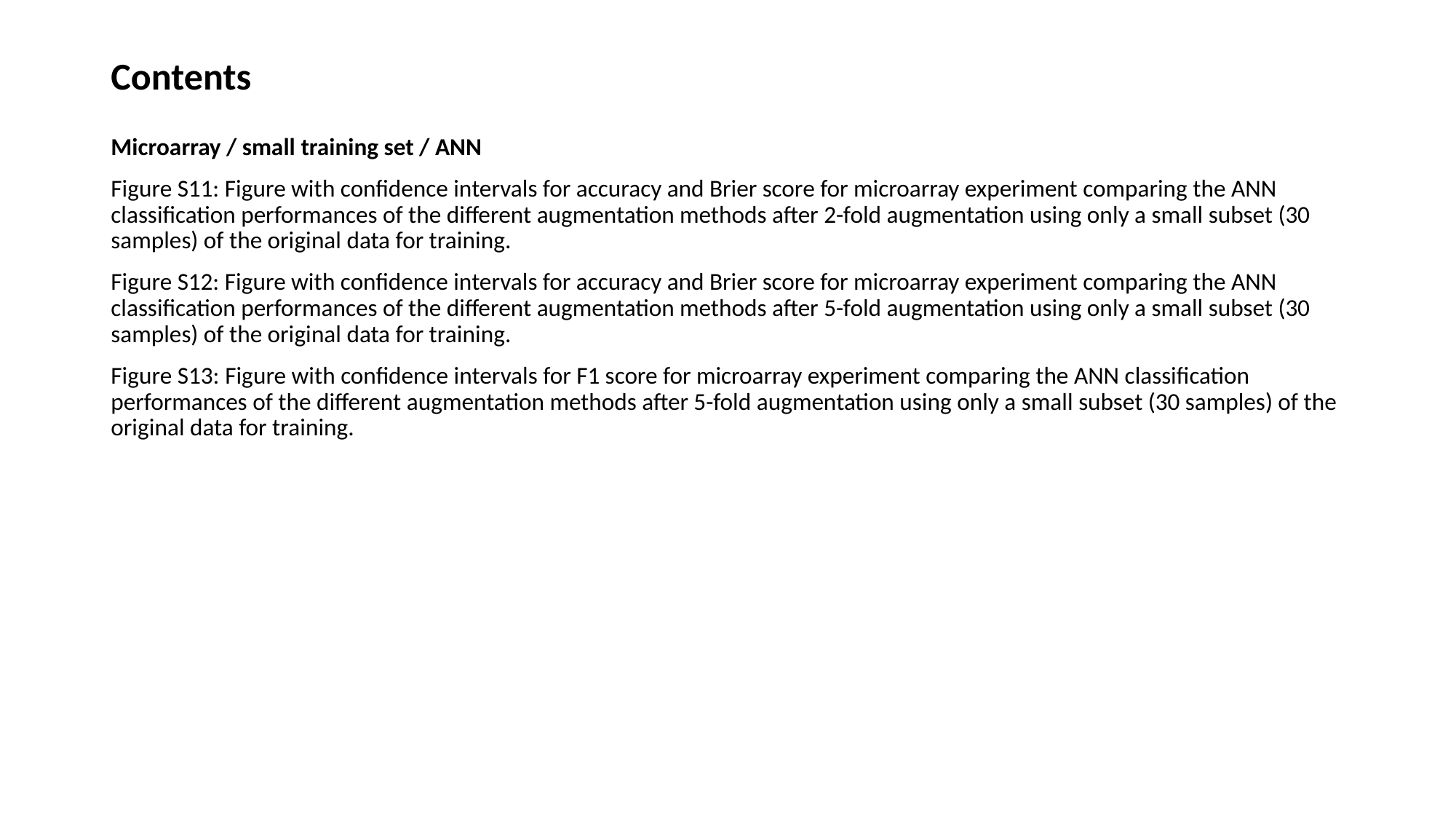

# Contents
Microarray / small training set / ANN
Figure S11: Figure with confidence intervals for accuracy and Brier score for microarray experiment comparing the ANN classification performances of the different augmentation methods after 2-fold augmentation using only a small subset (30 samples) of the original data for training.
Figure S12: Figure with confidence intervals for accuracy and Brier score for microarray experiment comparing the ANN classification performances of the different augmentation methods after 5-fold augmentation using only a small subset (30 samples) of the original data for training.
Figure S13: Figure with confidence intervals for F1 score for microarray experiment comparing the ANN classification performances of the different augmentation methods after 5-fold augmentation using only a small subset (30 samples) of the original data for training.

## Slide 4
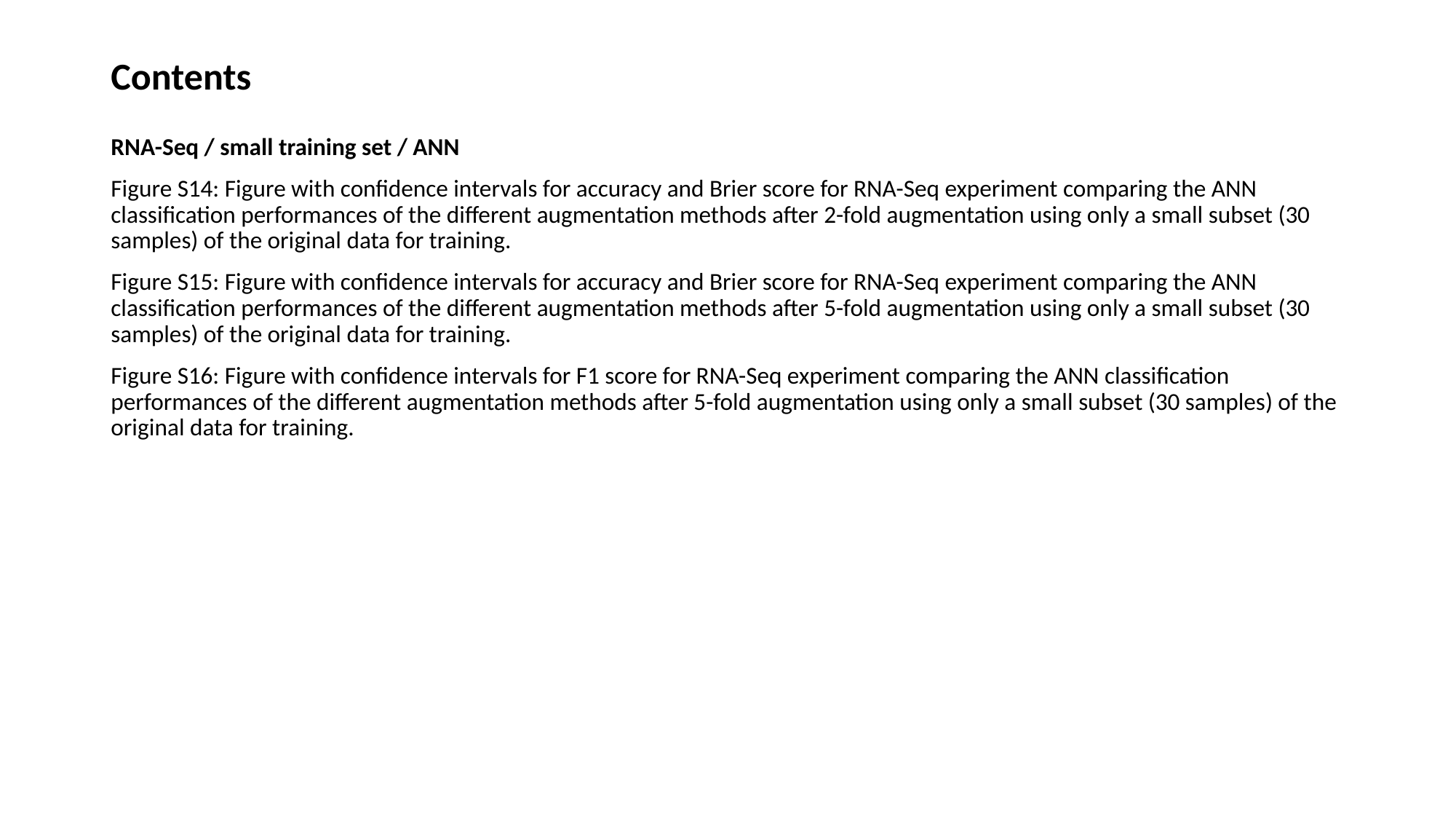

# Contents
RNA-Seq / small training set / ANN
Figure S14: Figure with confidence intervals for accuracy and Brier score for RNA-Seq experiment comparing the ANN classification performances of the different augmentation methods after 2-fold augmentation using only a small subset (30 samples) of the original data for training.
Figure S15: Figure with confidence intervals for accuracy and Brier score for RNA-Seq experiment comparing the ANN classification performances of the different augmentation methods after 5-fold augmentation using only a small subset (30 samples) of the original data for training.
Figure S16: Figure with confidence intervals for F1 score for RNA-Seq experiment comparing the ANN classification performances of the different augmentation methods after 5-fold augmentation using only a small subset (30 samples) of the original data for training.

## Slide 5
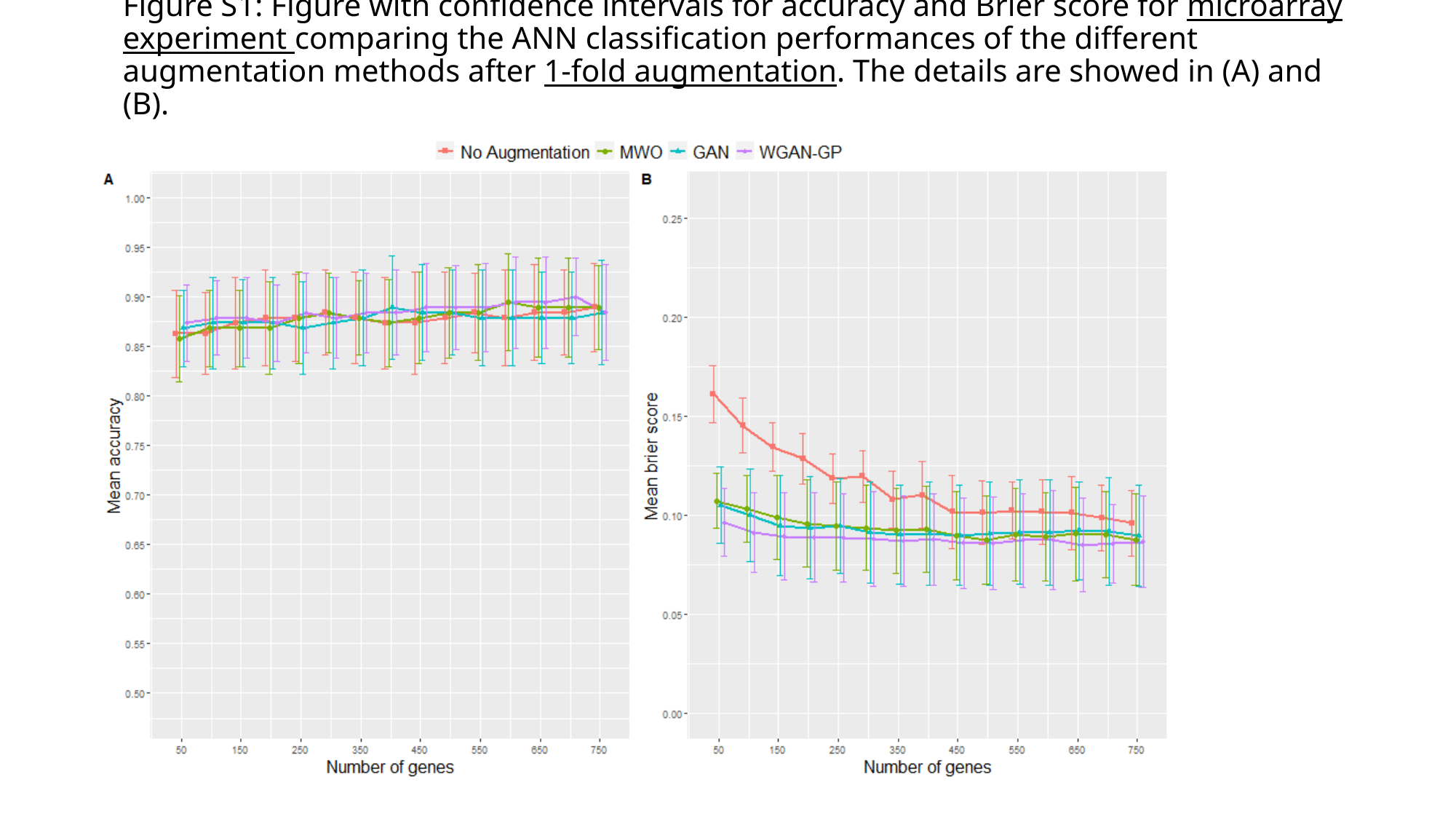

# Figure S1: Figure with confidence intervals for accuracy and Brier score for microarray experiment comparing the ANN classification performances of the different augmentation methods after 1-fold augmentation. The details are showed in (A) and (B).

## Slide 6
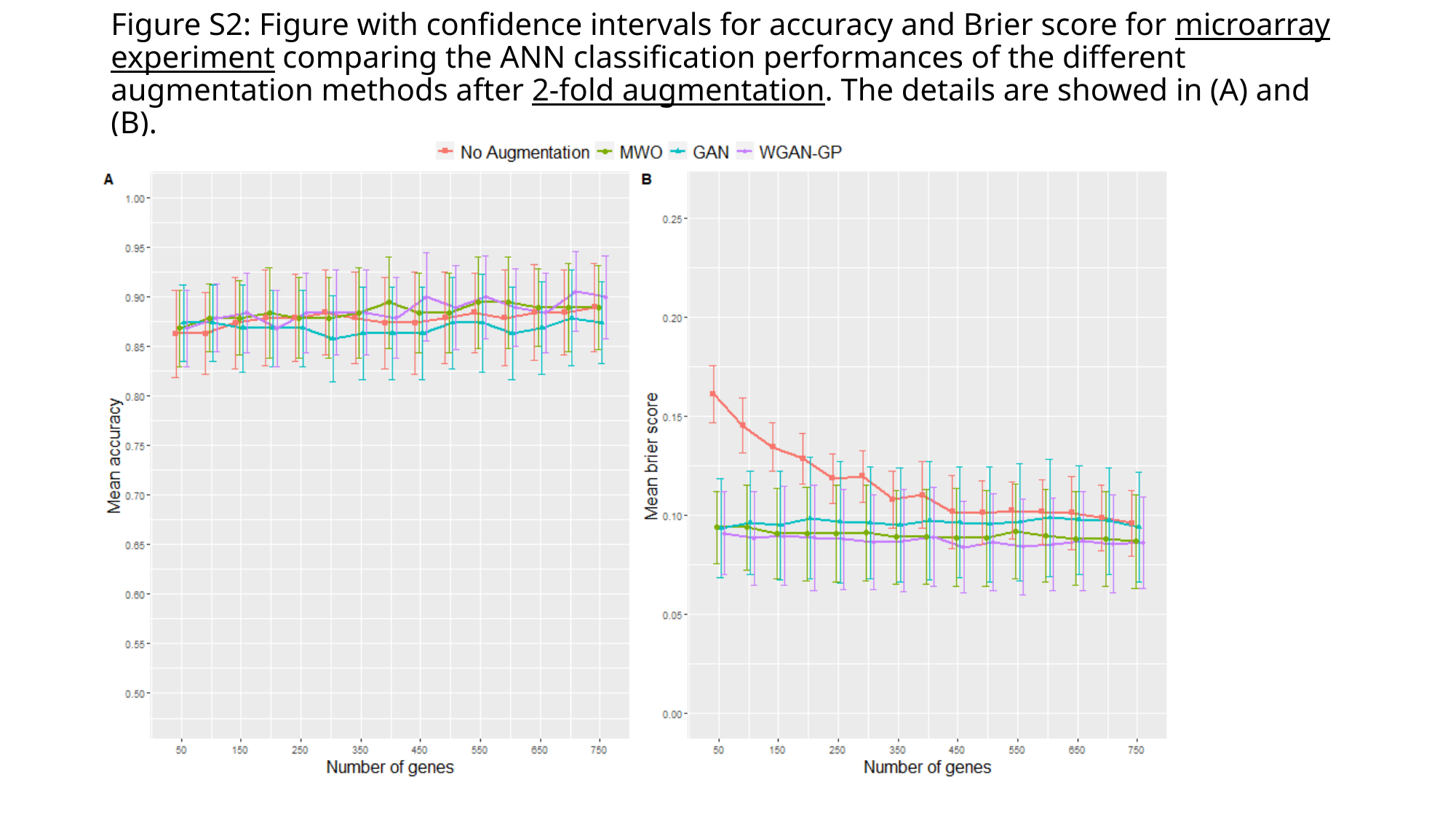

# Figure S2: Figure with confidence intervals for accuracy and Brier score for microarray experiment comparing the ANN classification performances of the different augmentation methods after 2-fold augmentation. The details are showed in (A) and (B).

## Slide 7
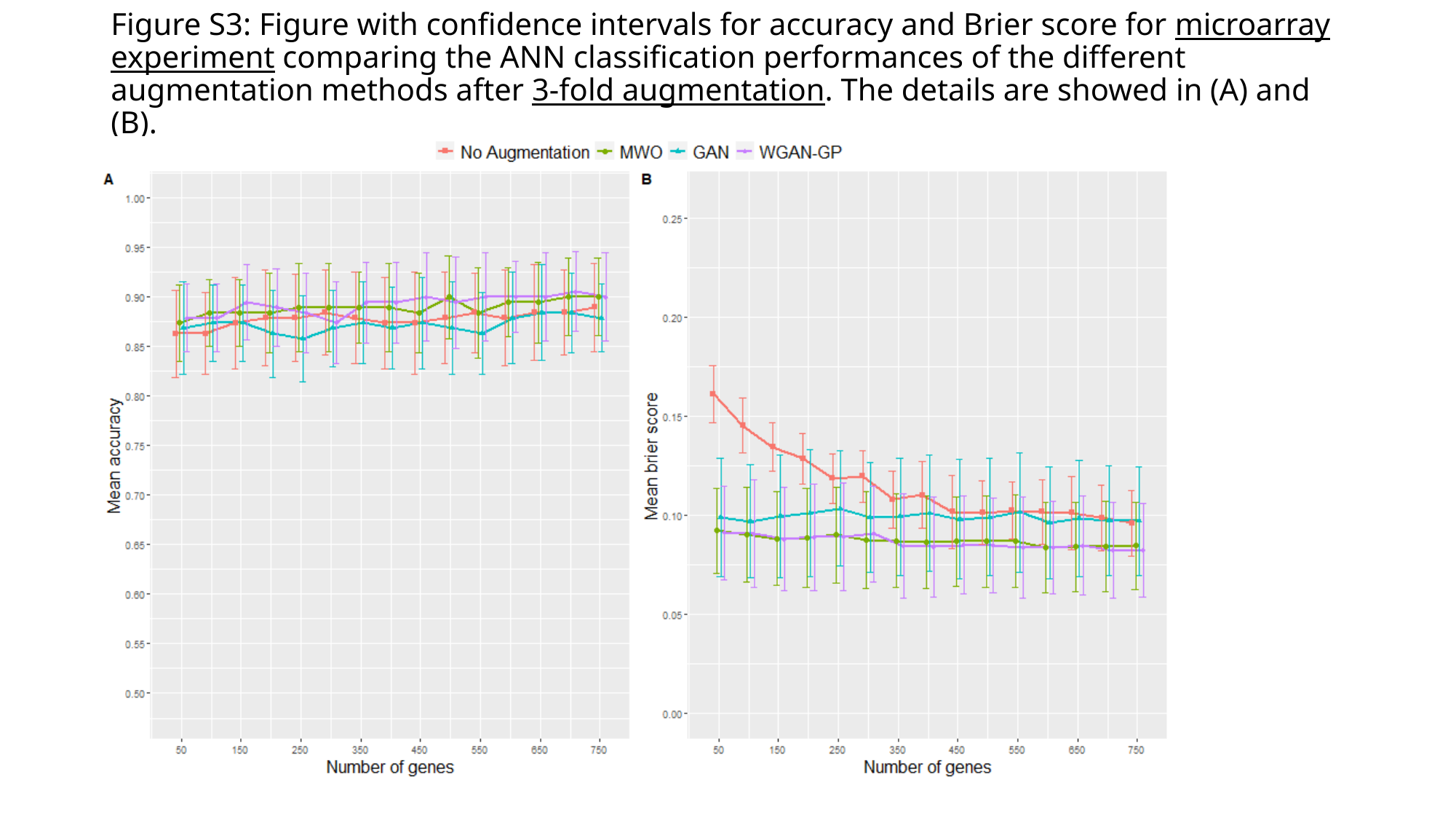

# Figure S3: Figure with confidence intervals for accuracy and Brier score for microarray experiment comparing the ANN classification performances of the different augmentation methods after 3-fold augmentation. The details are showed in (A) and (B).

## Slide 8
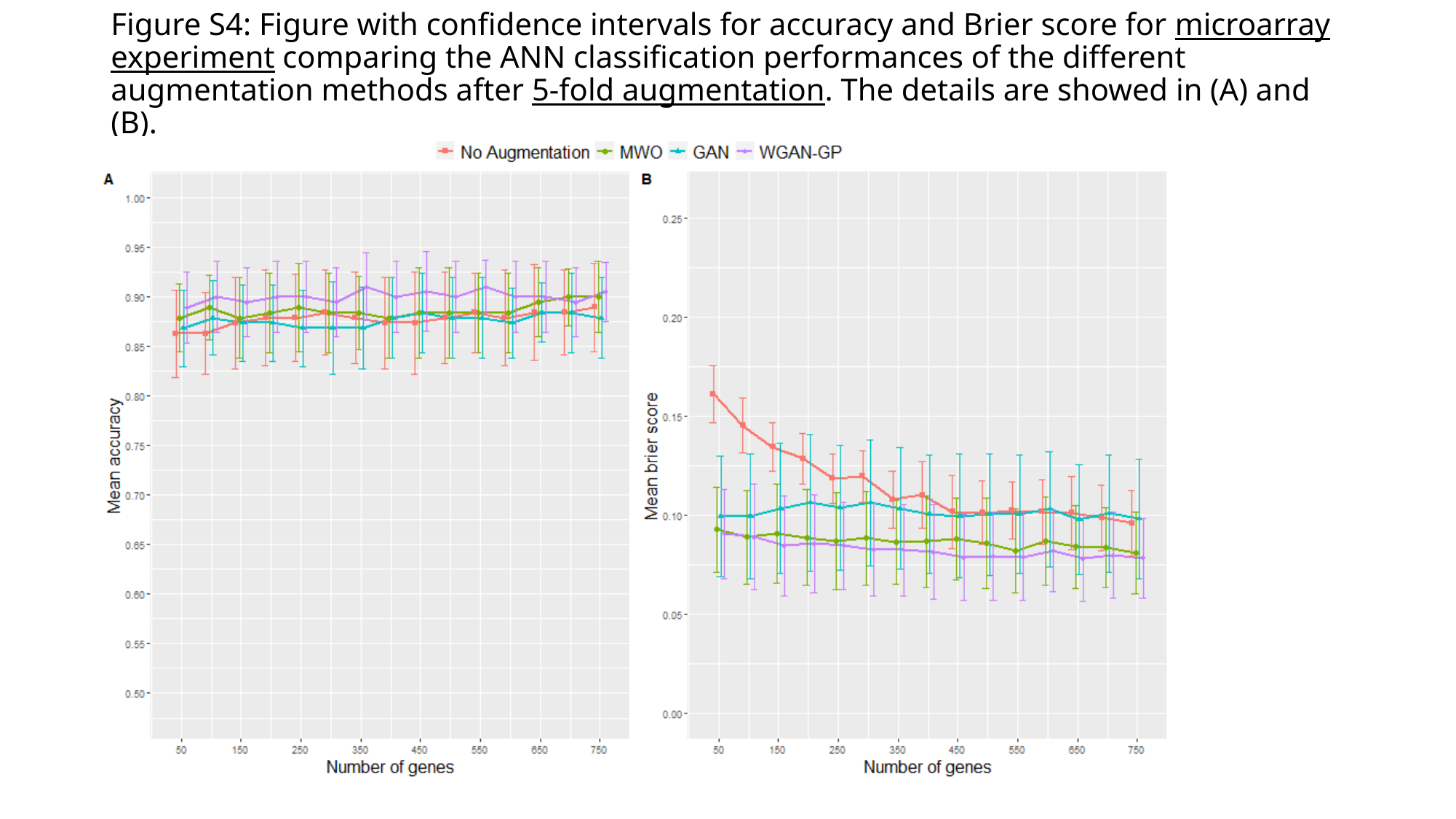

# Figure S4: Figure with confidence intervals for accuracy and Brier score for microarray experiment comparing the ANN classification performances of the different augmentation methods after 5-fold augmentation. The details are showed in (A) and (B).

## Slide 9
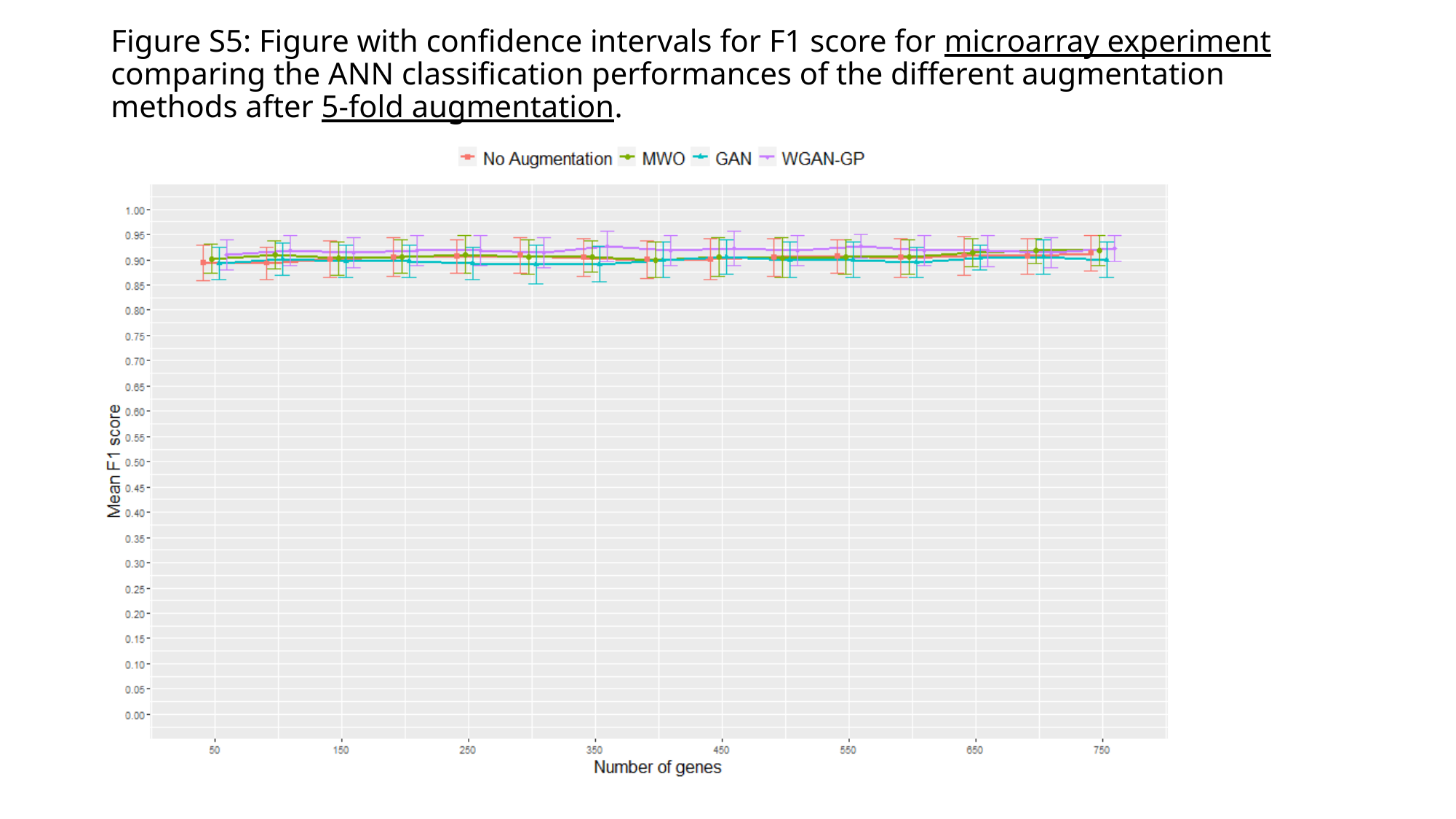

# Figure S5: Figure with confidence intervals for F1 score for microarray experiment comparing the ANN classification performances of the different augmentation methods after 5-fold augmentation.

## Slide 10
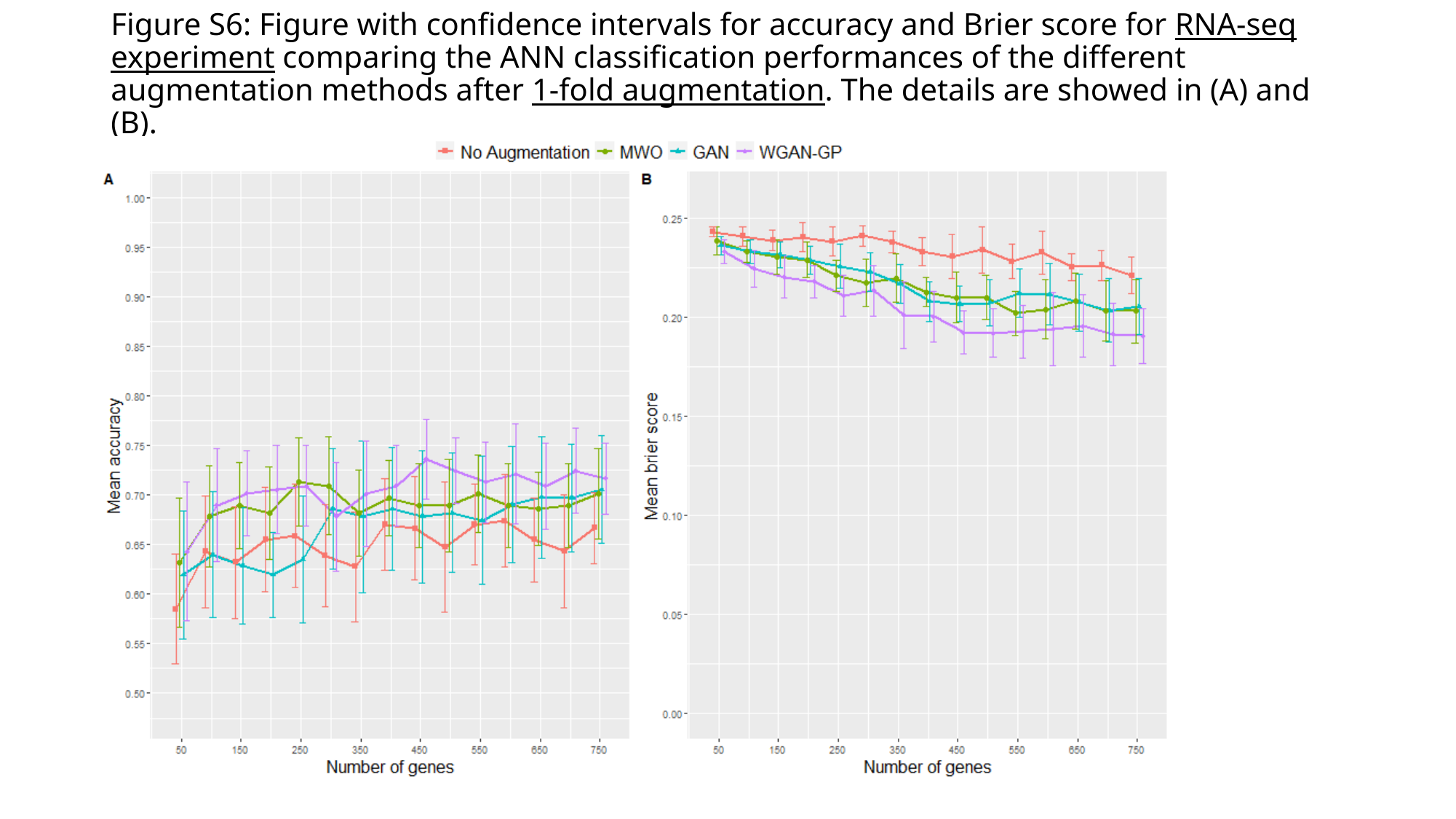

# Figure S6: Figure with confidence intervals for accuracy and Brier score for RNA-seq experiment comparing the ANN classification performances of the different augmentation methods after 1-fold augmentation. The details are showed in (A) and (B).

## Slide 11
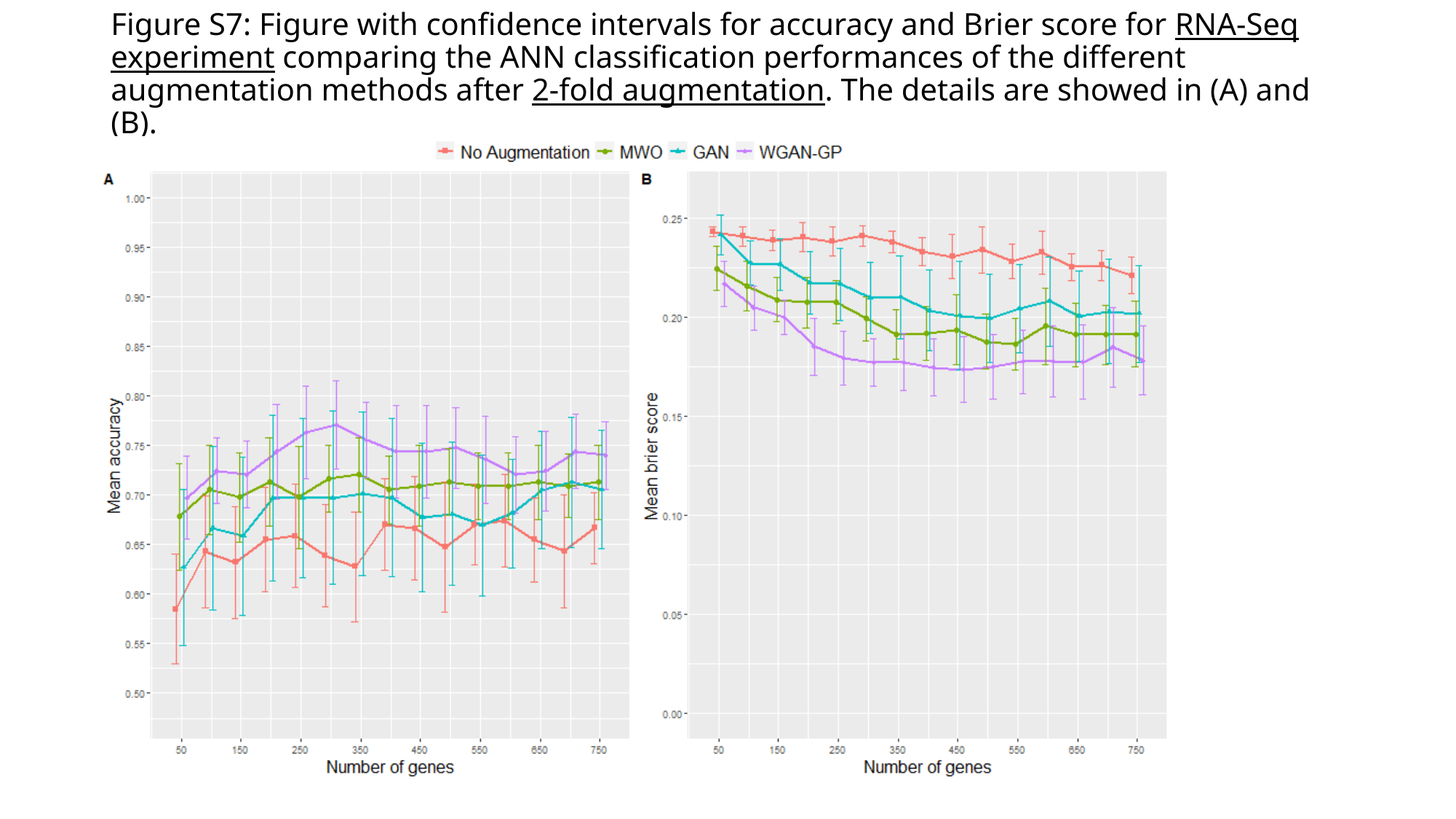

# Figure S7: Figure with confidence intervals for accuracy and Brier score for RNA-Seq experiment comparing the ANN classification performances of the different augmentation methods after 2-fold augmentation. The details are showed in (A) and (B).

## Slide 12
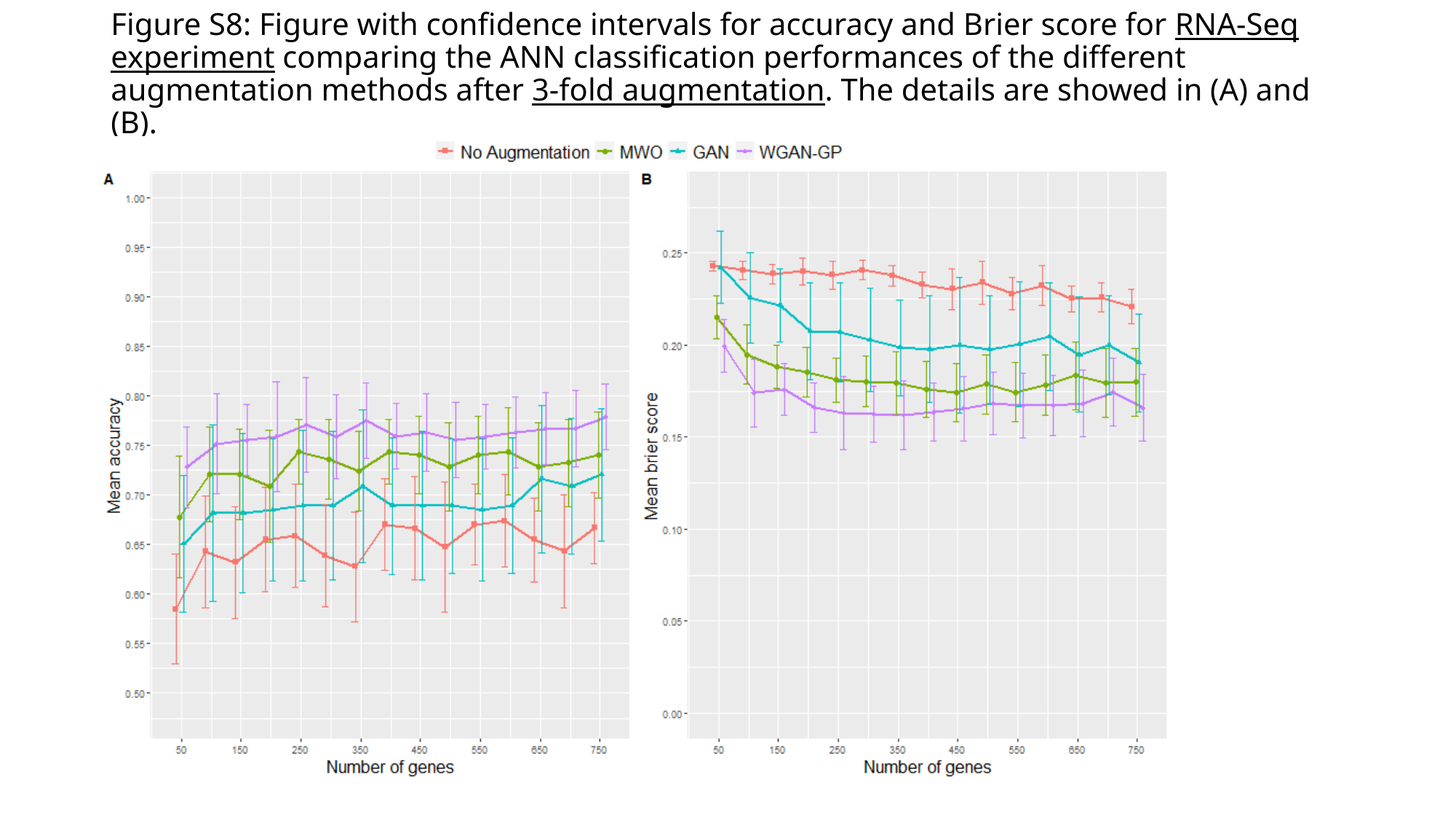

# Figure S8: Figure with confidence intervals for accuracy and Brier score for RNA-Seq experiment comparing the ANN classification performances of the different augmentation methods after 3-fold augmentation. The details are showed in (A) and (B).

## Slide 13
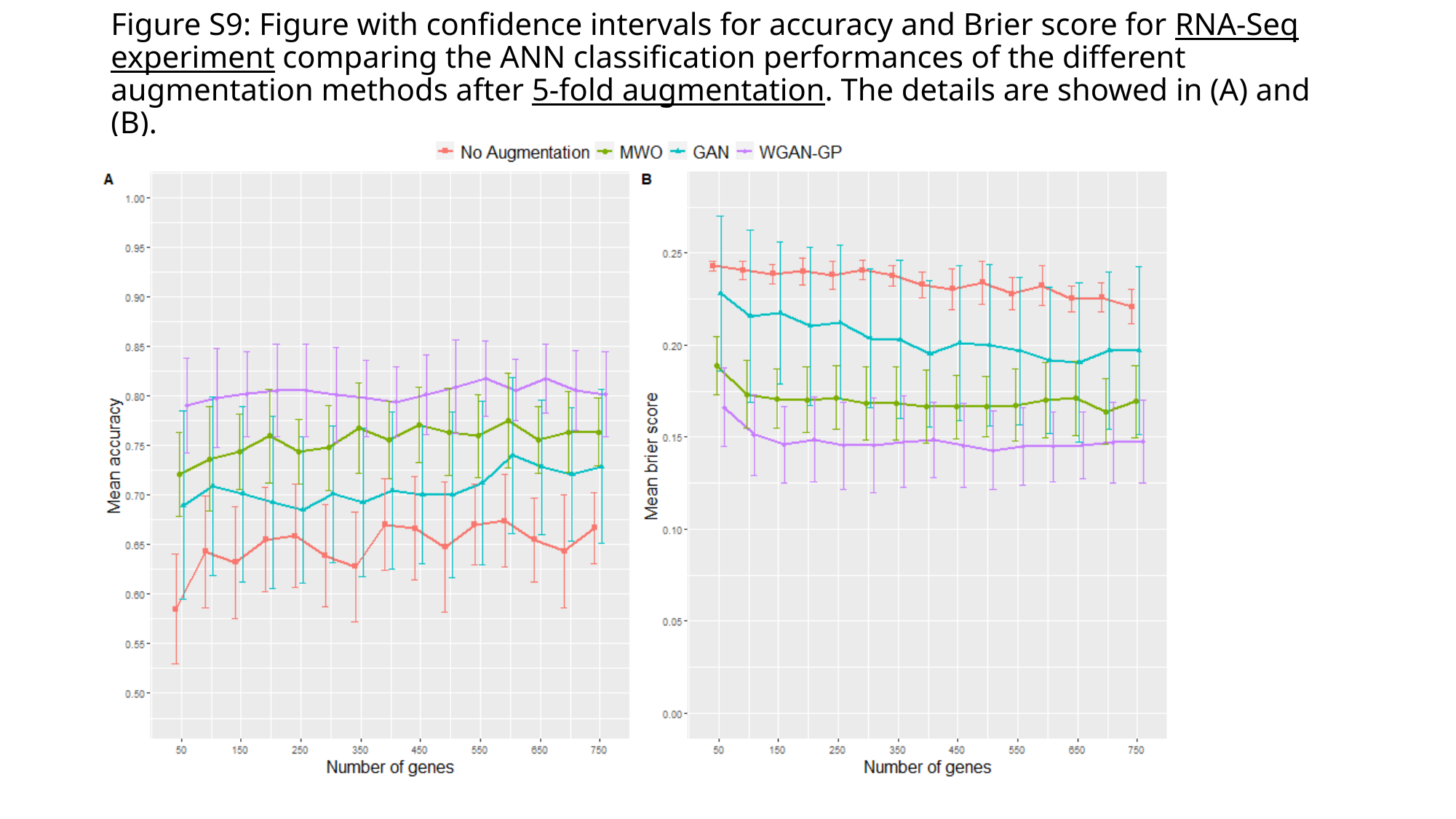

# Figure S9: Figure with confidence intervals for accuracy and Brier score for RNA-Seq experiment comparing the ANN classification performances of the different augmentation methods after 5-fold augmentation. The details are showed in (A) and (B).

## Slide 14
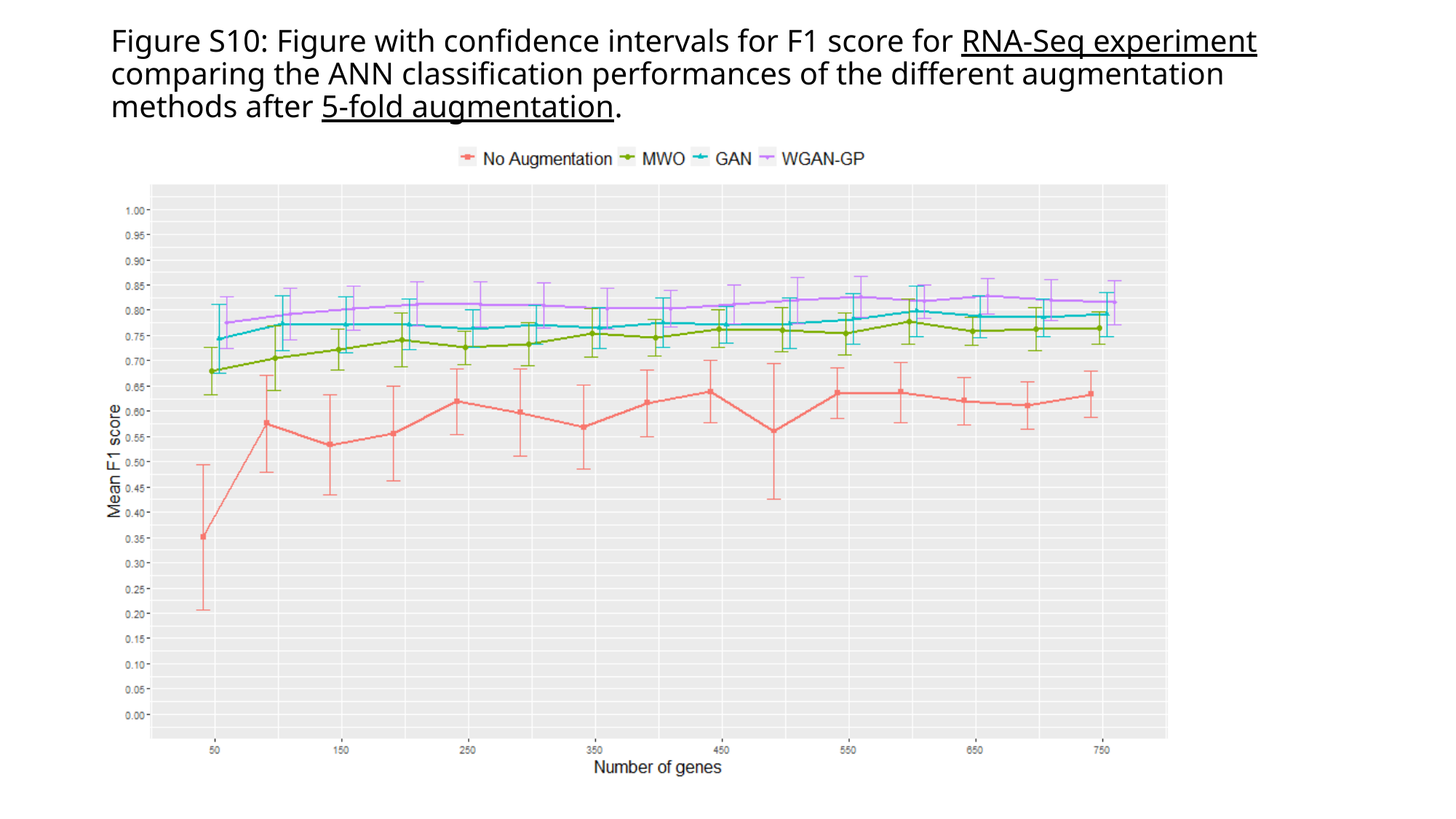

# Figure S10: Figure with confidence intervals for F1 score for RNA-Seq experiment comparing the ANN classification performances of the different augmentation methods after 5-fold augmentation.

## Slide 15
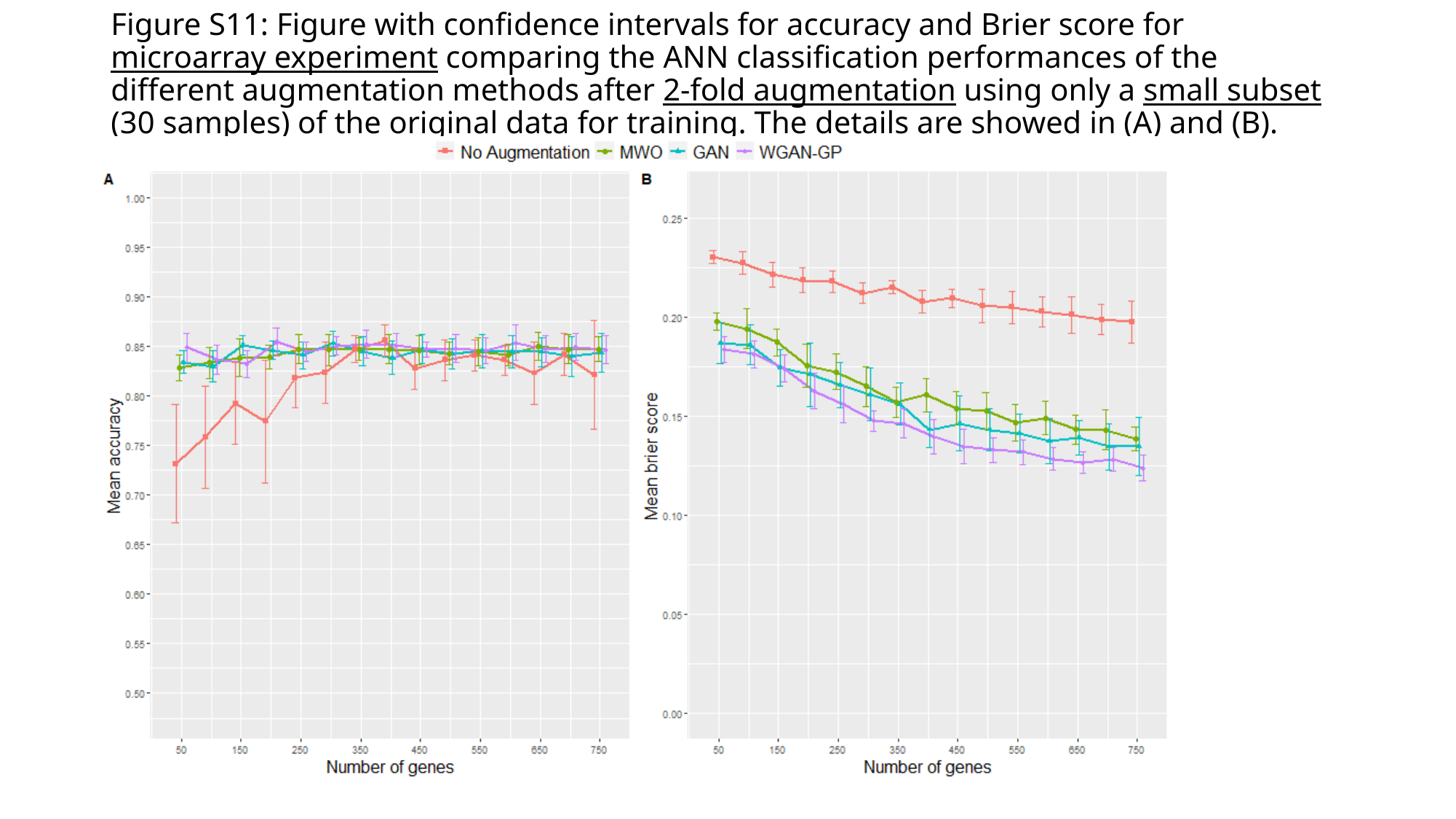

# Figure S11: Figure with confidence intervals for accuracy and Brier score for microarray experiment comparing the ANN classification performances of the different augmentation methods after 2-fold augmentation using only a small subset (30 samples) of the original data for training. The details are showed in (A) and (B).

## Slide 16
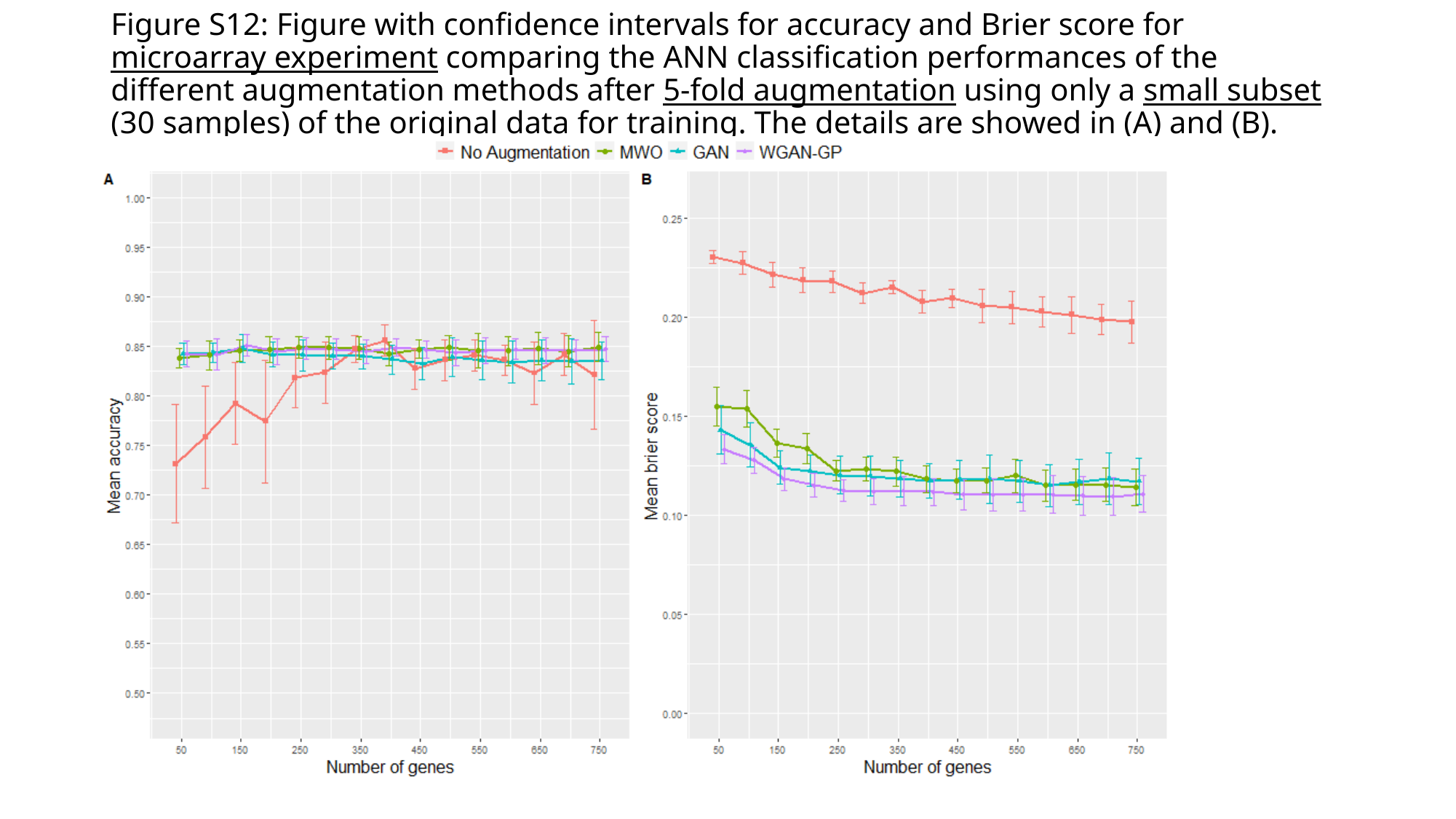

# Figure S12: Figure with confidence intervals for accuracy and Brier score for microarray experiment comparing the ANN classification performances of the different augmentation methods after 5-fold augmentation using only a small subset (30 samples) of the original data for training. The details are showed in (A) and (B).

## Slide 17
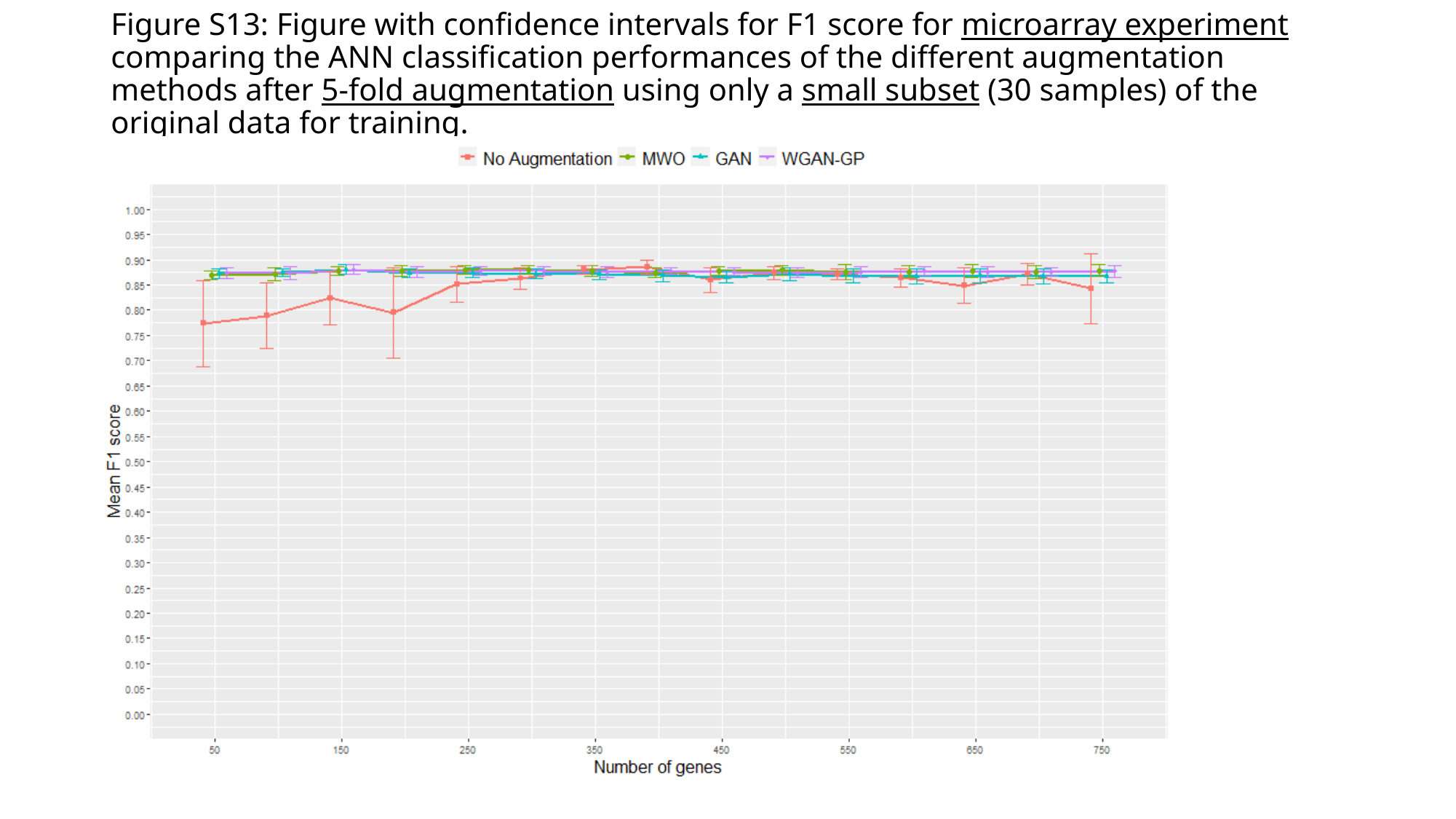

# Figure S13: Figure with confidence intervals for F1 score for microarray experiment comparing the ANN classification performances of the different augmentation methods after 5-fold augmentation using only a small subset (30 samples) of the original data for training.

## Slide 18
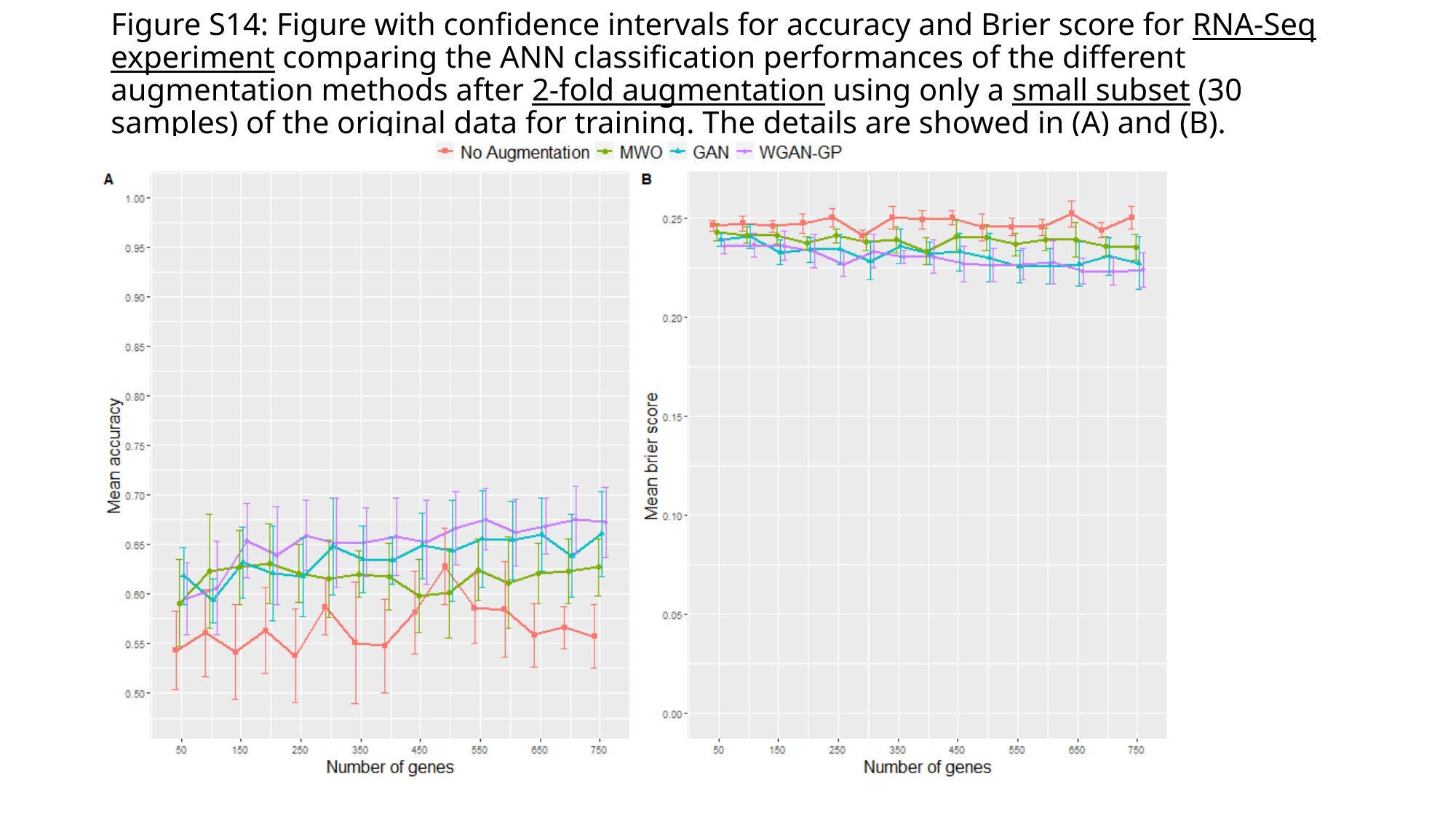

# Figure S14: Figure with confidence intervals for accuracy and Brier score for RNA-Seq experiment comparing the ANN classification performances of the different augmentation methods after 2-fold augmentation using only a small subset (30 samples) of the original data for training. The details are showed in (A) and (B).

## Slide 19
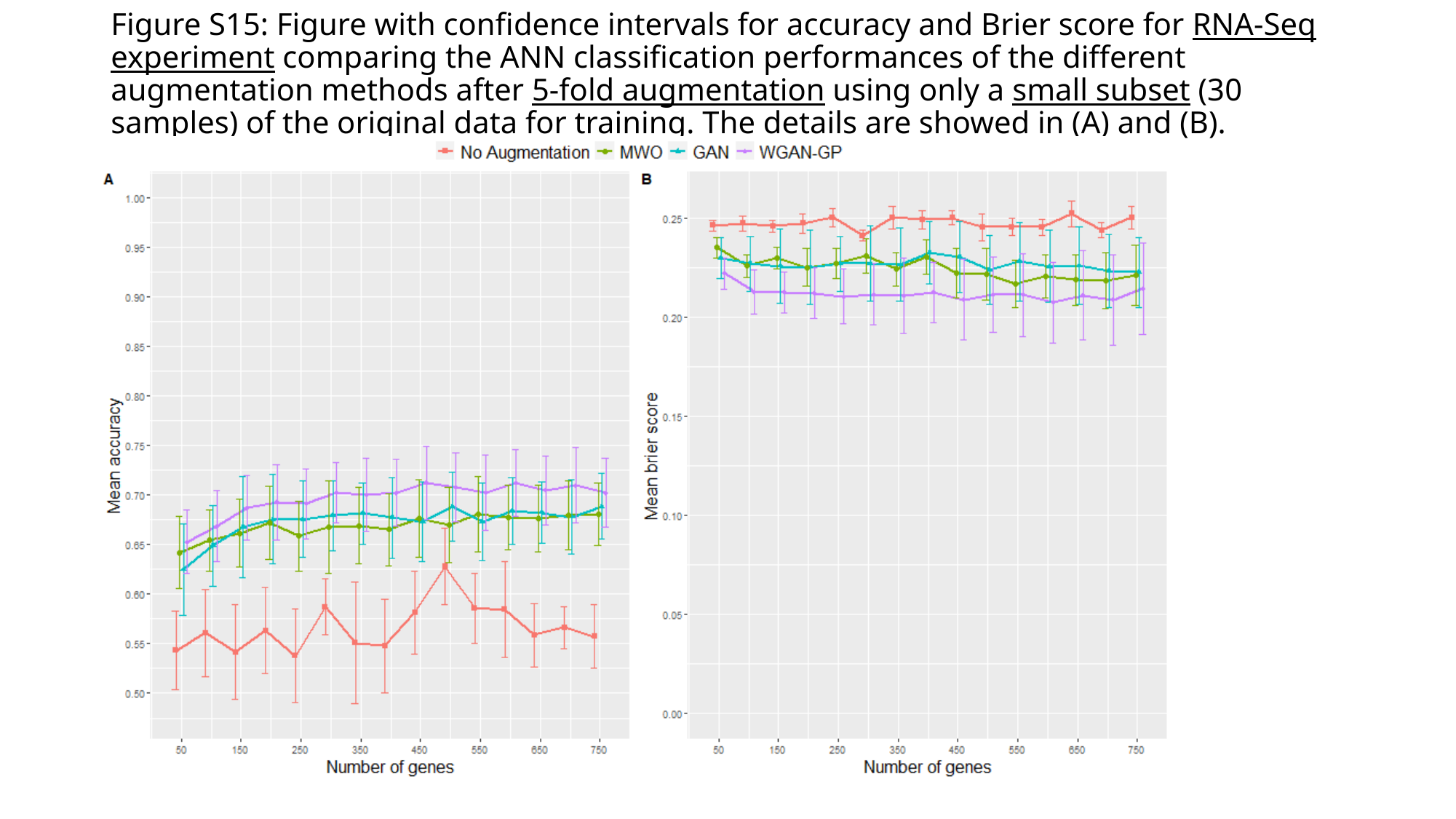

# Figure S15: Figure with confidence intervals for accuracy and Brier score for RNA-Seq experiment comparing the ANN classification performances of the different augmentation methods after 5-fold augmentation using only a small subset (30 samples) of the original data for training. The details are showed in (A) and (B).

## Slide 20
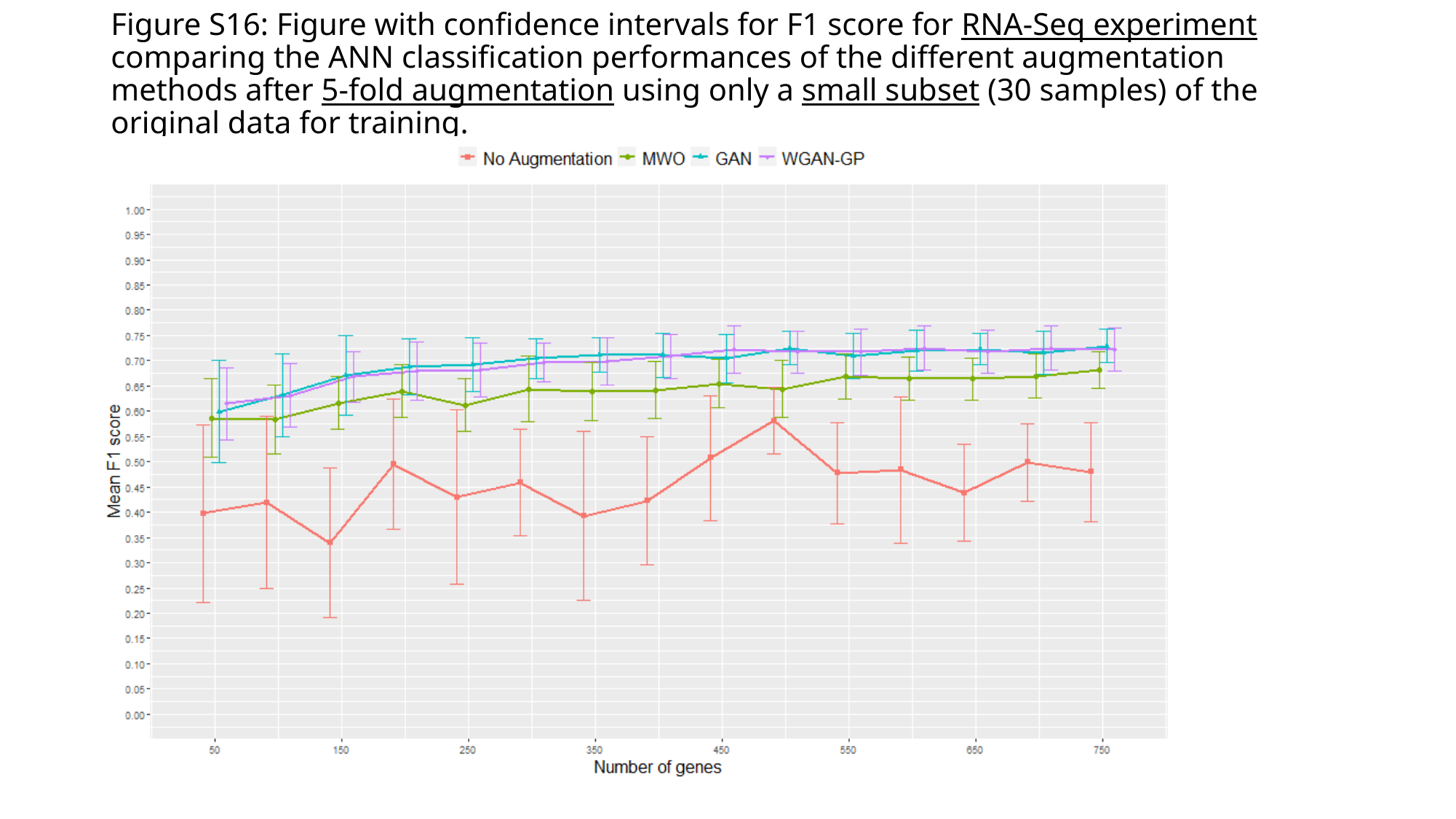

# Figure S16: Figure with confidence intervals for F1 score for RNA-Seq experiment comparing the ANN classification performances of the different augmentation methods after 5-fold augmentation using only a small subset (30 samples) of the original data for training.
